# Functional analysis of germline *VANGL2* variants using rescue assays of *vangl2* knockout zebrafish

**DOI:** 10.1093/hmg/ddad171

**Published:** 2023-10-10

**Authors:** Christopher J Derrick, Emmanuelle Szenker-Ravi, Adrian Santos-Ledo, Ahlam Alqahtani, Amirah Yusof, Lorraine Eley, Alistair H L Coleman, Sumanty Tohari, Alvin Yu-Jin Ng, Byrappa Venkatesh, Essa Alharby, Luke Mansard, Marie-Noelle Bonnet-Dupeyron, Anne-Francoise Roux, Christel Vaché, Joëlle Roume, Patrice Bouvagnet, Naif A M Almontashiri, Deborah J Henderson, Bruno Reversade, Bill Chaudhry

**Affiliations:** Biosciences Institute, Newcastle University, International Centre for Life, Central Parkway, Newcastle upon Tyne NE1 3BZ, United Kingdom; Genome Institute of Singapore (GIS), A*STAR, 60 Biopolis St, 138672, Singapore; Biosciences Institute, Newcastle University, International Centre for Life, Central Parkway, Newcastle upon Tyne NE1 3BZ, United Kingdom; Biosciences Institute, Newcastle University, International Centre for Life, Central Parkway, Newcastle upon Tyne NE1 3BZ, United Kingdom; Genome Institute of Singapore (GIS), A*STAR, 60 Biopolis St, 138672, Singapore; Biosciences Institute, Newcastle University, International Centre for Life, Central Parkway, Newcastle upon Tyne NE1 3BZ, United Kingdom; Biosciences Institute, Newcastle University, International Centre for Life, Central Parkway, Newcastle upon Tyne NE1 3BZ, United Kingdom; Institute of Molecular and Cell Biology, A*STAR, 61 Biopolis Dr, Proteos, 138673, Singapore; Institute of Molecular and Cell Biology, A*STAR, 61 Biopolis Dr, Proteos, 138673, Singapore; MGI Tech Singapore Pte Ltd, 21 Biopolis Rd, 138567, Singapore; Institute of Molecular and Cell Biology, A*STAR, 61 Biopolis Dr, Proteos, 138673, Singapore; Center for Genetics and Inherited Diseases, Taibah University, 7534 Abdul Muhsin Ibn Abdul Aziz, Al Ihn, Al-Madinah al-Munawwarah 42318, Saudi Arabia; Faculty of Applied Medical Sciences, Taibah University, Janadah Bin Umayyah Road, Tayba, Al-Madinah al-Munawwarah 42353, Saudi Arabia; Molecular Genetics Laboratory, University of Montpellier, CHU Montpellier, 163 Rue Auguste Broussonnet, 34090 Montpellier, France; Institute for Neurosciences of Montpellier (INM), University of Montpellier, Inserm, 80 Av. Augustin Fliche, 34000 Montpellier, France; Department of Genetics, Valence Hospital's Center, 179 Bd Maréchal Juin, 26000 Valence, France; Molecular Genetics Laboratory, University of Montpellier, CHU Montpellier, 163 Rue Auguste Broussonnet, 34090 Montpellier, France; Institute for Neurosciences of Montpellier (INM), University of Montpellier, Inserm, 80 Av. Augustin Fliche, 34000 Montpellier, France; Molecular Genetics Laboratory, University of Montpellier, CHU Montpellier, 163 Rue Auguste Broussonnet, 34090 Montpellier, France; Institute for Neurosciences of Montpellier (INM), University of Montpellier, Inserm, 80 Av. Augustin Fliche, 34000 Montpellier, France; Département de Génétique, CHI Poissy, St Germain-en-Laye, 10 Rue du Champ Gaillard, 78300 Poissy, France; CPDPN, Hôpital MFME, CHU de Martinique, Fort de France, Fort-de-France 97261, Martinique, France; Center for Genetics and Inherited Diseases, Taibah University, 7534 Abdul Muhsin Ibn Abdul Aziz, Al Ihn, Al-Madinah al-Munawwarah 42318, Saudi Arabia; Faculty of Applied Medical Sciences, Taibah University, Janadah Bin Umayyah Road, Tayba, Al-Madinah al-Munawwarah 42353, Saudi Arabia; Biosciences Institute, Newcastle University, International Centre for Life, Central Parkway, Newcastle upon Tyne NE1 3BZ, United Kingdom; Genome Institute of Singapore (GIS), A*STAR, 60 Biopolis St, 138672, Singapore; Institute of Molecular and Cell Biology, A*STAR, 61 Biopolis Dr, Proteos, 138673, Singapore; Smart-Health Initiative, BESE, KAUST, Thuwal, 23955-6900, Kingdom of Saudi Arabia; Medical Genetics Department, Koç Hospital Davutpaşa Caddesi 34010 Topkapı Istanbul, Istanbul, Turkey; Biosciences Institute, Newcastle University, International Centre for Life, Central Parkway, Newcastle upon Tyne NE1 3BZ, United Kingdom

**Keywords:** zebrafish, variant of unknown significance, planar cell polarity, congenital heart defect, neural tube defect

## Abstract

Developmental studies have shown that the evolutionarily conserved Wnt Planar Cell Polarity (PCP) pathway is essential for the development of a diverse range of tissues and organs including the brain, spinal cord, heart and sensory organs, as well as establishment of the left-right body axis. Germline mutations in the highly conserved PCP gene *VANGL2* in humans have only been associated with central nervous system malformations, and functional testing to understand variant impact has not been performed. Here we report three new families with missense variants in *VANGL2* associated with heterotaxy and congenital heart disease p.(Arg169His), non-syndromic hearing loss p.(Glu465Ala) and congenital heart disease with brain defects p.(Arg135Trp). To test the *in vivo* impact of these and previously described variants, we have established clinically-relevant assays using mRNA rescue of the *vangl2* mutant zebrafish. We show that all variants disrupt Vangl2 function, although to different extents and depending on the developmental process. We also begin to identify that different *VANGL2* missense variants may be haploinsufficient and discuss evidence in support of pathogenicity. Together, this study demonstrates that zebrafish present a suitable pipeline to investigate variants of unknown significance and suggests new avenues for investigation of the different developmental contexts of VANGL2 function that are clinically meaningful.

## Introduction

Congenital malformations arise due to failures in developmental processes and are a leading cause of mortality and morbidity throughout life. Sequencing of patients to identify potential genetic causes for congenital heart disease (CHD) and neural tube defects (NTDs), the two most common birth-defects [1, 2] which arise during early embryogenesis, have identified mutations in genes referred to as variants of unknown significance (VUS) [3–6].

One pathway of particular developmental importance is the evolutionarily conserved Wnt Planar Cell Polarity (Wnt-PCP) pathway [7]. Wnt-PCP signalling establishes proximo-distal identity within epithelial sheets and coordinates polarized cell behaviours during development through asymmetric localization of core components including Frizzled (Fzd) and Dishevelled (Dvl) at the distal membrane and the Van Gogh-Like proteins (Vangl1 and Vangl2) at the proximal membrane [8]. Additionally, the pathway can respond to secreted non-canonical Wnt ligands to alter cellular behaviours through modulation of cytoskeletal components [9]. Animal studies have shown that disruption to PCP signalling impacts multiple developmental processes, resulting in severe defects across the embryo, including the brain, spinal cord, heart, gut and sensory organs [10–13]. Homozygous *Vangl2* mutations in *Loop-tail* (*Lp*) mice and the *trilobite* (*tri*) zebrafish result in a shortened body axis due to the loss of positional information necessary to drive convergent extension (CE) at gastrulation [14, 15]. Subsequently, and independent of earlier embryonic events, Vangl2 is required both in mice and zebrafish for caudal migration of facial branchiomotor (nVII) neurons (which innervate muscles in the vertebrate head) from rhombomere 4 (r4) to rhombomere 6 (r6) [14, 16–20]. *Lp* mice also show NTDs [21, 22], cardiac malformations, [23] together with abnormalities of cilial patterning and function in multiple structures [24–26]. PCP signalling is necessary for the positioning of both sensory and motile cilia during development, with the orientation of fields of cilia randomized in PCP mutants including *Vangl2* in both mice and zebrafish [26, 27]. Cilia of the inner ear are required for hearing and balance [26, 28] whilst those in the lateral line system of fish detect water movements [29]. Cilia also function in the left–right organizer (LRO) to ensure the TGF-β ligand *Nodal* (*southpaw*, *spaw* in zebrafish) is asymmetrically expressed in the left lateral plate mesoderm (LPM) [30, 31] where it is proposed to instruct the asymmetric morphogenesis of the organ anlagen [32]. Disordered positioning and/or function of cilia within the LRO can cause laterality disturbances leading to mispositioned organs across the left–right axis (heterotaxy). Heterotaxy occurs in 1 in 10 000 live births in humans and is frequently associated with CHD [33, 34]. Independently of laterality defects, the loss of *Vangl2* in mice results in a spectrum of cardiovascular malformations [23] and loss of *vangl2* in zebrafish also results in abnormal heart morphogenesis [35].

**Figure 1:**
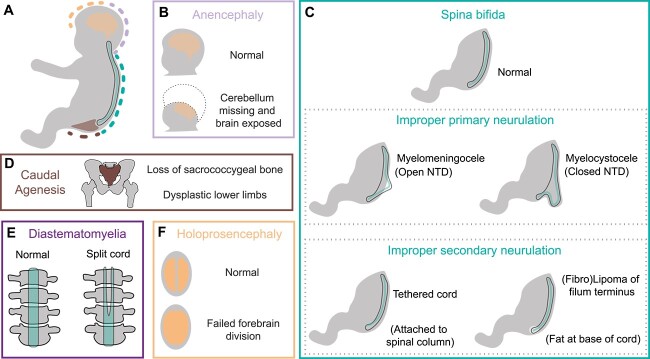
Congenital malformations associated with *VANGL2* coding sequence missense mutations. (A) Regions associated with abnormal development in patients carrying heterozygous coding sequence mutations in *VANGL2*. (B) Anencephaly, failure of anterior neural tube closure during primary neurulation, leading to loss of cerebellum and open cranial cavity. (C) Different forms of spina bifida arising due to failure of primary neurulation which can result in an open or closed spinal column. *VANGL2* patients also display failures in secondary neurulation: tethered cord, where free movement of the spinal cord within the column is restricted, and lipoma at the base of the spinal cord (filum terminus). (D) Caudal agenesis (also known as caudal regression syndrome or sacral agenesis) is defined by the loss of the sacrococcygeal bone (brown), associated with dysplastic lower limbs and impacts other caudally located organs. (E) Diastematomyelia is the splitting of the spinal cord. (F) Failure of division of the embryonic forebrain results in holoprosencephaly.

Heterozygous missense mutations in the *VANGL2* coding sequence have been identified in patients and foetuses with a variety of neurological and spinal defects (Fig. 1). Failure of primary neurulation results in the most severe NTDs, which in *VANGL2* patients are anencephaly (Fig. 1B), myelomeningocele and myelocystocele (Fig. 1C). Defects arising from improper secondary neurulation in patients heterozygous for *VANGL2* include cord tethering and lipoma of the filum terminus (Fig. 1C). Other developmental defects of the lumbar-sacral region such as caudal agenesis (Fig. 1D) and diastematomyelia (Fig. 1E) have been associated with *VANGL2* variants and are thought to arise from defective gastrulation, although some studies have linked caudal agenesis to improper secondary neurulation [36, 37]. *VANGL2* has also been linked to holoprosencephaly (Fig. 1F) a malformation distinct from NTDs. Despite these case reports, a meta-analysis has not supported a strong relationship between *VANGL2* variants and NTD risk [38]. Furthermore, in these studies some parents of the affected individuals also carried the same missense variant, yet did not present with any clear clinical symptoms [39] and missense *VANGL2* variants have also been identified in healthy control populations [39]. Separately, large genomic studies of CHD patients have failed to identify individuals carrying variants in *VANGL2* or other core PCP pathway members. More generally, given the pleiotropic effects in *Lp* mice and *vangl2* zebrafish [14, 15, 23, 40–43], it is surprising that associations between disruption to *VANGL2*, or more generally, PCP activity, have not been made with more phenotypically severe, recessive human developmental syndromes. Hence, understanding the relevance of these *VANGL2* variants and VUS generally remains a major challenge.

There has been agreement on the classification of variants in genes known to cause Mendelian disorders [44], however the processes for allocating variants outside of strong syndromic or pedigree information is less clear. Bioinformatic algorithms predicting missense variant impact are 65%–80% accurate when examining known disease-causing genes, but are recognized as having poor specificity, leading to over-reporting of variants as deleterious [45]. Furthermore, it is important to differentiate causative pathogenic variants from variants predicted to be disruptive or damaging but not necessarily implicated in a specific disease [44]. Hence, objective functional assays are of immense value in characterizing the relevance of variants [46, 47]. Although *in vitro* methods are attractive in ascribing functional impact of variants [47], understanding developmental disorders necessitates animal-based assays [48]. Although mice currently provide the closest tractable laboratory model for human disease, a balance must be struck between a model with human developmental relevance, whilst ensuring sufficiently high throughput. Zebrafish have been suggested as a good model system to examine VUS, as over 70% of disease-causing genes are conserved with humans [49]. Furthermore, their rapid, extrauterine development, in a temporally reproducible fashion, which is highly amenable to genetic manipulation, allows for a semi high-throughput approach [48, 50]. However, zebrafish present some complications such as a whole genome duplication event and evolutionary differences in peptide structure [49, 51]. With regard to *vangl2*, only a single copy is found in the zebrafish genome and there is a high level of biochemical and functional conservation, with human *VANGL2* mRNA acting similarly to zebrafish *vangl2* mRNA [52].

In this study, we examine whether the zebrafish embryo is suitable for *VANGL2* variant analysis for some of the different roles that *vangl2* mediates in organogenesis, through mRNA rescue assays of the well-characterized *vangl2* mutant. We evaluate previously published VUS and two novel variants linked with families affected by CHD and deafness. We show that all examined *VANGL2* VUS are potentially deleterious, but to varying extents in different biological processes, that may be further modified by the presence of a single functional copy of the gene. Overall, this work begins to identify the developmental basis for some clinical phenotypes associated with *VANGL2* variants, highlighting the importance of using appropriate functional assays when assessing variants of unknown significance.

## Materials and Methods

### Patient variant isolation

*Family 14*: Duo WES studies for the parents due to the lack of patients samples were indicated based on the phenotype and family history to identify the causal variants in known or candidate genes. DNA was extracted from whole blood samples collected in EDTA tubes. The DNA libraries were prepared and sequenced using the SureSelect Kit (Agilent, Santa Clara, CA, USA) and Hiseq2000 platform (Illumina, San Diego, CA, USA), respectively. The Genome Analysis Toolkit (GATK) was used for variant calling. Variants in known and candidate genes were classified as per the ACMG guidelines. Allele frequencies were verified using the genome aggregation (gnomAD) and Saudi Human Genome Project (SHGP) databases [53]. Sanger sequencing was performed as previously described [54].

*Family 15*: Genomic DNA (gDNA) was extracted from either whole blood or foetal tissue. DNA concentration and quality were assessed using NanoDrop (Thermo Scientific) and Qubit (Life technologies) fluorometers. A260/A280 ratios of 1.8 to 2.0 and A260/A230 ratios > 1.5 were accepted. DNA fragmentation was assessed using agarose gel (0.8%) electrophoresis. High-quality gDNA (1 μg) of both affected individuals was used for exome capture with the ION TargetSeq Exome Kit. The exome library was prepared on an ION OneTouch System and sequenced on an Ion Proton instrument (Life Technologies) using a one ION PI chip. Sequence reads were aligned to the human reference genome (Human GRCh37 (hg19) build) using the Torrent Mapping Alignment Program (TMAP) from the Torrent Suite (v.5.0.2). The variants were called using the Torrent Variant Caller (TVC) plugin (v.5.0.2) and were annotated with the associated gene, location, protein position and amino acid changes, quality-score, coverage, predicted functional consequences using SIFT [55], PolyPhen2 [56], Grantham [57] prediction scores, phyloP [58] conservation scores, and 5000 genomes Minor Allele Frequencies. Variants were filtered for common SNPs using the NCBI’s “common and no known medical impacts” database, the Exome Aggregate Consortium (http://exac.broadinstitute.org/) and the Exome Sequencing Project (http://evs.gs.washington.edu/EVS/). We only kept variants that were common between the two affected siblings. We next removed variants that were present in greater than 1% of the previously in-house 465 sequenced individuals. A total of 17.4 Gb and 17.7 Gb were sequenced with an average read length of 187 bp and 189 bp for individuals F15-II:1 and F15-II:2, respectively. An average coverage of 201× and 198× was achieved over the exome, with 96% of bases covered at least 20× for each individual. A combined total of 60 106 variants were identified across protein-coding exons, UTRs, splice sites and flanking introns. Additional filters were applied to retain variants that were homozygous or heterozygous in both probands. A final set of 306 variants remained, of which 136 were in exonic regions. The heterozygous c.506G > A; p.(Arg169His) missense variant in *VANGL2* was of interest due to its role in left–right asymmetry establishment. Subsequently, the parents were tested for variant segregation analysis using the following primers to amplify *VANGL2* exon 4, forward: 5′-CCAAGGACATGGAGGACAGT-3′ and reverse: 5′-ATGGCCAACGTTGTAGAAGC-3′.

*Family 16*: Genomic DNA was obtained from blood samples belonging to the proband and his family. Library preparation was performed with the Nimbelgen SeqCap EZ MedExome kit (Roche Technology) following the manufacturer’s instructions. Exome-enriched libraries were sequenced using the Illumina NextSeq system (Illumina, San Diego, CA, USA). Bioinformatic analysis of sequencing data was realized using an in-house pipeline (https://github.com/beboche/nenufaar) to generate a merged BAM and VCF file for the family. Quality data revealed more than 97.8% of the target nucleotides covered at 30× with a mean coverage of more than 120× for the four individuals. Tertiary analysis involved the MobiDL captainAchab workflow (https://github.com/mobidic/MobiDL), based on ANNOVAR [59], MPA [60] and Captain-ACHAB (https://github.com/mobidic/Captain-ACHAB). Variants were considered of interest if all the hearing-impaired patients analysed carried the same variant, with a control population allele frequency (gnomAD v2) of 0.3% or less. All variants already classed as benign or likely benign in clinvar were automatically excluded. Candidate gene was considered if meeting the following criteria: expression in inner ear cells (SHIELD, https://shield.hms.harvard.edu/), interaction with other proteins known to be involved in hearing loss (literature review), or reported zebrafish/mice strains with HL (https://zfin.org/;  https://www.informatics.jax.org/). Familial segregation of the candidate variants was then performed using Sanger sequencing following classical methodology.

**Table 1:** Historic cases of germline variants in coding sequence of *VANGL2*.

Family	Main clinical phenotype	Reference	Method of identification	Zygosity	Nucleotide change	Protein change	gnomAD allele frequency	Polyphen	SIFT	CADD score
1	Holoprosencephaly	Lei et al., 2010	*VANGL2* sequenced	Heterozygous	c.251C > T (not c.737C > T)	S84F	Never seen	Probably damaging	Deleterious	26.6
2	Control	Kibar et al., 2011	*VANGL2* sequenced	Heterozygous	c.313C > T	R105C	3.98e-5	Probably damaging	Deleterious	26.4
3	Myelomenigocele	Kibar et al., 2011	*VANGL2*, *DVL2* and *DVL3* sequenced	Heterozygous^a^	c.403C > T	R135W	8.90e-5	Probably damaging	Deleterious	28.2
4	Diastematomyelia	Kibar et al., 2011	*VANGL2*, *DVL2* and *DVL3* sequenced	Heterozygous	c.530G > A	R177H	never seen	Probably damaging	Deleterious	28.7
5	Control	Kibar et al., 2011	*VANGL2* sequenced	Heterozygous	c.532G > A	V178I	6.39e-5	Benign	Tolerated	19.15
6	Tethered cord		*VANGL2*, *DVL2* and *DVL3* sequenced							
7	Myelocystocele	Kibar et al., 2011	*VANGL2*, *DVL2* and *DVL3* sequenced	Heterozygous	c.724C > G	L242V	6.03e-4	Benign	Deleterious	25.5
8	Myelomenigocele		*VANGL2*, *DVL2* and *DVL3* sequenced							
9	Lipoma of filum terminus	Kibar et al., 2011	*VANGL2*, *DVL2* and *DVL3* sequenced	Heterozygous	c.740C > T	T247M	2.96e-5	Possibly damaging	Tolerated	26.6
10	Fibrolipoma of filum terminus	Kibar et al., 2011	*VANGL2*, *DVL2* and *DVL3* sequenced	Heterozygous	c.809G > A	R270H	1.06e-5	Probably damaging	Deleterious	29.7
11	Anencephaly, spina bifida	Lei et al., 2010	*VANGL2* sequenced	Heterozygous	c.1057C > T (not c.1543C > T)	R353C	7.97e-6	Probably damaging	Deleterious	32
12	Anencephaly	Lei et al., 2010	*VANGL2* sequenced	Heterozygous	c.1310 T > C (not c.1796 T > C)	F437S	Never seen	Probably damaging	Deleterious	28.9
13	Caudal agenesis	Kibar et al., 2011	*VANGL2*, *DVL2* and *DVL3* sequenced	Heterozygous^a^	c.1445G > A	R482H	1.10e-4	Benign	Tolerated	22.8

Table summarizing frequency and *in silico* predictions of impact of *VANGL2* missense variants. A CADD score great than 25 is defined at potentially pathogenic.

^a^Patients also carrying a heterozygous mutation in *DVL2*. See also Supplementary Table 1.

**Table 2:** New families with germline variants in *VANGL2*.

Family	Main clinical phenotype	Method of identification	Zygosity	Nucleotide change	Protein change	gnomAD allele frequency	Polyphen	SIFT	CADD score
14	New disease	Duo exome	Homozygous?	c.403C > T	R135W	8.90e-5	Probably damaging	Deleterious	28.2
15	Heterotaxy	Duo exome	Heterozygous	c.506G > A	R169H	never seen	Probably damaging	Deleterious	31
16	Hearing loss	Duo exome	Heterozygous	c.1394A > C	E465A	2.33e-4	Possibly damaging	Deleterious	27

Table summarizing frequency and *in silico* predictions of impact of *VANGL2* missense variants. A CADD score great than 25 is defined at potentially pathogenic. See also Supplementary Table 1.

### Variant frequency and pathogenicity predictions

Variant frequencies shown in Tables 1 and 2 were obtained from the genome Aggregation Database (gnomAD) v2.1.1 [61]. Variant pathogenicity predictions were conducted using the PolyPhen2 [56], SIFT [55] and Combined annotation-dependent depletion (CADD) scores [62] open access softwares.

### Generation of human *VANGL2* containing plasmids and mRNA *in vitro* transcription

Heart tissue was lysed in TRIzol reagent (Ambion 15596026) and total RNA purified following standard protocols. cDNA was synthesized using Superscript III (Invitrogen 18080-044).

The *VANGL2* coding sequence was isolated from human cDNA by a standard GoTaq G2 (Promega M27845) PCR using primers: Forward: 5′-**GGGCCCACC**ATGGACACCGAGTCCCAGTA-3′ (initiating codon underlined, Kozak sequence and ApaI restriction site in bold) and Reverse: 5′-**CTCGAG**TCACACTGAGGTCTCAGACT-3′ (termination codon underlined, XhoI restriction site in bold) and cloned into pCR8/GW/TOPO (ThermoFisher K250020) to create the middle entry vector pME-HuVANGL2. The pME-HuVANGL2 was combined with p5E-pCS2+, p3E-poly(A) and pDestTol2pA3 in an LR gateway reaction [63], using LR Clonase II (ThermoFisher 11791020) to generate the WT *VANGL2* plasmid.

Single nucleotide changes were introduced into the WT *VANGL2* plasmid with the Quick Lightning Kit (Agilent) by primers given in Supplementary Table 8.

All plasmids underwent Sanger sequencing (Eurofins genomics) to confirm absence of PCR errors and introduction of correct alterations. Template plasmid was linearized overnight by KpnI (NEB R3142) digest and cleaned up by using the Qiagen PCR Purification Kit. Approximately 1 μg of linearized template was used in an *in vitro* transcription reaction with the Sp6 mMessage Machine Kit and capped mRNA was cleaned up using Ammonium Acetate precipitation (ThermoFisher AM1340).

RNA was diluted to a stock concentration (1 μg/nl) and stored long term at −80°C, or further diluted to a working concentration (200 ng/nl) and kept at −20°C. To minimize freeze/thaw cycles, RNA was defrosted at most twice.

### Zebrafish husbandry and micro-injection

The following, previously described lines were used: *vangl2^m209^* (formerly *trilobite^m209^*) [14, 41, 64] and *Tg(isl1a:GFP)^rw0^* [65]. Both lines were maintained in an AB background.

Embryos obtained from natural pairwise mating of heterozygous *vangl2^m209^* adults were injected at the 1-cell stage. Phenol Red (Sigma P0290) was used as a tracer in all injection mixes to ensure that uninjected embryos were not included in analysis. Embryos were maintained at 28.5°C in E3 medium (5 mM NaCl, 0.17 mM KCl, 0.33 mM CaCl_2_, 0.33 mM MgSO_4_) and staged according to Kimmel [66]. At ~6 hpf, unfertilized or dead embryos were removed prior to analysis. Analysis of heart tube morphology and positioning and *spaw* expression were carried out in embryos obtained from the same injection session. Other assays were carried out independently. For analysis of *spaw* expression, development of injected embryos was slowed overnight and restaged according to Kimmel [66]; otherwise, embryos were raised at 28.5°C. For each assay, mRNA injection was carried out at least twice in distinct clutches of embryos.

Embryos older than 24 hpf were transferred into E3 medium containing 0.003% 1-phenyl-2-thiourea (PTU, Sigma P7629) to prevent pigment formation, aiding imaging. Embryos were fixed in 4% Paraformaldehyde (PFA, P6148 Sigma) in 1× Phosphate Buffered Saline (PBS, Oxoid BR0014G) overnight at 4°C.

### *vangl2^m209^* genotyping

The *m209* allele is a T > A transversion in intron 7 (ENSDART00000033316.5: g.46988 T > A) resulting in a 13 bp insertion of intronic sequence, leading to a frameshift with a premature stop codon [15]. It can be identified using dCAPS [67] following PCR amplification using primers: Forward 5′-CAGCCTTTATACTCTCTTCCATTGG-3′ and Reverse 5′-CAGAAATGCCTGACCACAGATTC-3′ and digestion with AlwNI (NEB, R0514) for 3.5 h. The reverse primer (base change for dCAPs underlined) together with the *m209* mutation (3′ to the reverse primer binding site) introduces an AlwNI restriction site.

Genomic DNA (gDNA) was extracted in Single Embryo Lysate (SEL, 50 mM KCl, 2.5 mM MgCl_2_, 10 mM Tris pH 8.3, 0.005% NP40, 0.005% Tween-20, 0.001% Gelatine) with 100 μg/ml Proteinase K (Sigma, P2308) incubated at 60°C for 1 h then 95°C for 15 min. Extracted gDNA was diluted 1:1 with ddH_2_O and 2 μl used in a standard GoTaq G2 Polymerase PCR, with annealing temperature 62°C, 36 cycles and a 30 s extension time. Digested PCRs were resolved on a 4% Agarose TBE (89 mM Tris–HCl, 89 mM Boric Acid, 2 mM EDTA) gel.

### Analysis of convergent extension

At 24–28 hpf, embryos were manually dechorionated and lightly anaesthetized with MS22 (Tricaine). Embryos were positioned laterally and imaged live in 2% Methyl Cellulose (Sigma M0262) and then genotyped for *vangl2^m209^*. The freehand line tool in Fiji [68] was used to quantify the distance from the centre of the head to the end of the tail fin fold that followed the midline of the body (See Supplementary Fig. 2G).

### Analysis of branchiomotor neuron migration

Following fixation, embryos were washed 3 times in PBST (0.2% Tween 20 (Sigma P2287) in 1× PBS) at room temperature. The heads were dissected at the otic vesicle (leaving rhombomeres intact) and mounted in a 1:1 mix of VectaShield with DAPI (Vector labs H-1200) and PBST and imaged using Zeiss Axiotome microscope using a 20× objective. Images were blinded using the previously described Fiji plug-in [69]. The perpendicular distance between the caudal most point of an isl1a:GFP+ FBM neuron cell body and a straight line denoting where VII axons exited rhombomere 4, was defined as the maximum migration distance.

### Analysis of kinocilia orientation

Following fixation, embryos were rinsed 3 times in PBS at room temperature, blocked at room temperature for 1 h in BDPF (1% Bovine Serum Albumin (Sigma A2153), 1% DMSO (Merck, D4540), 5% Foetal Calf Serum (Fisher 11573397) in 1× PBS) and then incubated overnight at 4°C in BDPF with 1:200 Phalloidin 594 (Invitrogen A12381) [70]. Trunks were dissected and mounted in a 1:1 mix of VectaShield with DAPI and PBST. The left pL1 neuromast was identified by its positioning at somite 6/7 [71] and imaged using Nikon A1 inverted confocal with a 40× objective, 8× optical zoom and 0.225 μm step size. Quantifications and statistical analysis of cilia polarity were carried out as described in [27], samples were blinded prior to analysis [69].

### mRNA *in situ* hybridization

Following fixation, embryos were washed 3 times in PBST at room temperature and serially dehydrated into 100% MeOH for long term storage at −20°C. Whole mount *in situ* hybridization was carried out following standard protocols [72]. The following previously published probes were used: *spaw* [30], *myl7* [73].

### Analysis of early heart tube positioning and morphology

Scoring of heart tube positioning and presence of cardia bifida was performed live between 28–30 hpf [74]. Representative phenotypes are highlighted by mRNA *in situ* hybridization of the pan cardiac marker *myl7.*

### Statistical analysis of zebrafish rescue assays

For AP axis length and FBM neuron migration, each embryo was a single experimental unit, with sample size determined prior to injections and analysis. Injected embryos were obtained from at least 2 distinct injections sessions. For *spaw* expression and heart tube morphology, a clutch (each a population from a distinct injection session) was a single experimental unit, with sample size determined prior to injections and analysis. For neuromast polarity, orientations of 4–6 individual cilia from 8 separate embryos (from at least 2 distinct injections sessions) were pooled for overall analysis of AP or DV axis alignment; sample size was not predetermined. All statistical analyses were carried out in GraphPad Prism.

### Rescue definitions

A “full rescue” was defined by the ability of WT *VANGL2* mRNA injection into homozygous *vangl2^m209^* mutant embryos to generate a rescue that was statistically indistinguishable from the WT sibling phenotype (Figs 6B and 7B, D). A “good rescue” was defined by the ability of WT *VANGL2* mRNA injection into homozygous *vangl2^m209^* mutant embryos to generate a statistically significant rescue compared to injected homozygous *vangl2^m209^* mutant embryos, but was also significantly different from WT sibling embryos (Figs 4D and 5D).

For variant rescue analyses, each patient variant was compared to uninjected homozygous *vangl2^m209^* mutant and homozygous *vangl2^m209^* mutant injected with WT *VANGL2* mRNA. Where a given variants rescue activity was indistinguishable from WT *VANGL2* mRNA, the variant was defined as either “full rescue” or “good rescue” depending on efficacy of WT *VANGL2* mRNA rescue (see above). Where a given variant showed a statistically significant improvement compared to uninjected *vangl2^m209^* embryos, but was also significantly different from homozygous *vangl2^m209^* mutant embryos injected with WT *VANGL2* mRNA, the variant was defined as “some rescue”.

### Study approval

In Saudi Arabia, informed consents were obtained from all participating individuals (Family 14) as per an approved institutional review board (IRB) protocol (TU MLT-2019-07). For Family 15, the study protocol and genetic analyses performed in Singapore were approved by A^*^STAR IRB (2019-087). In France, the study has been approved by Institutional Review Board (IRB) of CHU de Montpellier: 2018_IRB-MTP_05-05 obtained on the 15 June 2018 (Family 16) and genetic analyses performed in France were done in accordance with bioethics rules of French law.

For isolation of human *VANGL2* cDNA, human embryonic heart tissue was provided by the Joint MRC/Wellcome Trust (Grants MR/006237/1, MR/X008304/1 and 226 202/Z/22/Z) Human Developmental Biology Resource (http://www.hdbr.org), with approval from the Newcastle and North Tyneside NHS Health Authority Joint Ethics Committee (18/NE/0290).

Adult zebrafish (*Danio rerio*) were maintained according to standard laboratory conditions and all procedures carried out in accordance with the local Animal Welfare and Ethical Review Body (AWERB), UK Home Office and Newcastle University (Project Licence P25F4F0F4).

## Results

### Previously reported *VANGL2* germline variants

**Figure 2:**
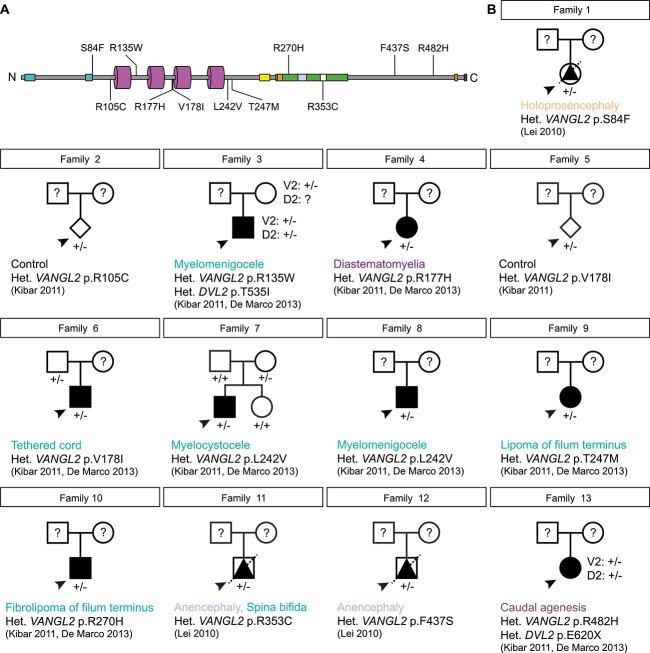
Collation of clinical data relating to historical *VANGL2* variants. (A) Primary structure of human VANGL2 (UniProt: Q9ULK5) [116, 117] showing known functional domains and location of VUS. Two N-terminal serine/threonine motifs (cyan [78]), 4 transmembrane domains (magenta, [98]), TGN (*trans*-Golgi network) sorting motif (yellow [118]), the Dishevelled and Prickle binding region (green [119–121], which overlaps with two ubiquitinoylation sites, (orange [122], a VCP interacting motif (mauve, [122])) and a nuclear localization signal (white [123]). Two further ubiquitinoylation sites (orange) and the Type I PDZ domain (blue, [124]) are present at the C-terminal end. (B) Clinical pedigrees, major developmental defects and original references relating to previously reported missense mutations in coding sequence of human *VANGL2*. Squares, circles, diamonds, triangles represent male, females, unknown gender, and foetuses, respectively. Arrowheads denote proband. Diagonal line denotes deceased. Open symbols: healthy, black symbols: affected. V2: *VANGL2* genotype, D2: *DVL2* genotype. Question mark denotes DNA unavailable. Het.: heterozygous.

There are 11 variants in 13 families previously reported to affect the *VANGL2* coding sequence (Fig. 2, Table 1, Supplementary Table 1). In all cases, heterozygous *VANGL2* mutations were identified by targeted sequencing of the locus [39, 75]. Of these 13 families, 8 probands, but not their parents (families 3, 4, 6–10 and 13, from Kibar *et al*. [39]) were also sequenced for *DVL2* and *DVL3* [76]. This identified two probands as digenic heterozygotes for *VANGL2* and *DVL2* (families 3 and 13).

The reported malformations in these 13 families were diverse, with only 5 (Families 3, 7, 8, 11 and 12) having NTDs (Figs 1B, C and 2). Tethering of the spinal cord was reported in family 6 and lipoma of the filum terminus in families 9 and 10 (Fig. 1C). Both diastematomyelia (split spinal cord) in family 4 (Fig. 1E) and caudal agenesis (absence of sacrococcygeal bone) in family 13 (Fig. 1D) are thought to arise from abnormal gastrulation, rather than neurulation. Holoprosencephaly, which has a distinct aetiology from NTDs, was reported in family 1 (Fig. 1F). In addition, *VANGL2* variants were identified in control and unaffected individuals (families 2, 3 and 5–7). Thus, there is considerable uncertainty about the true impact of these *VANGL2* variants during development, further complicated by the possibility of variable penetrance and variable expressivity.

### Novel *VANGL2* germline variants in families with multiple affected individuals

**Figure 3:**
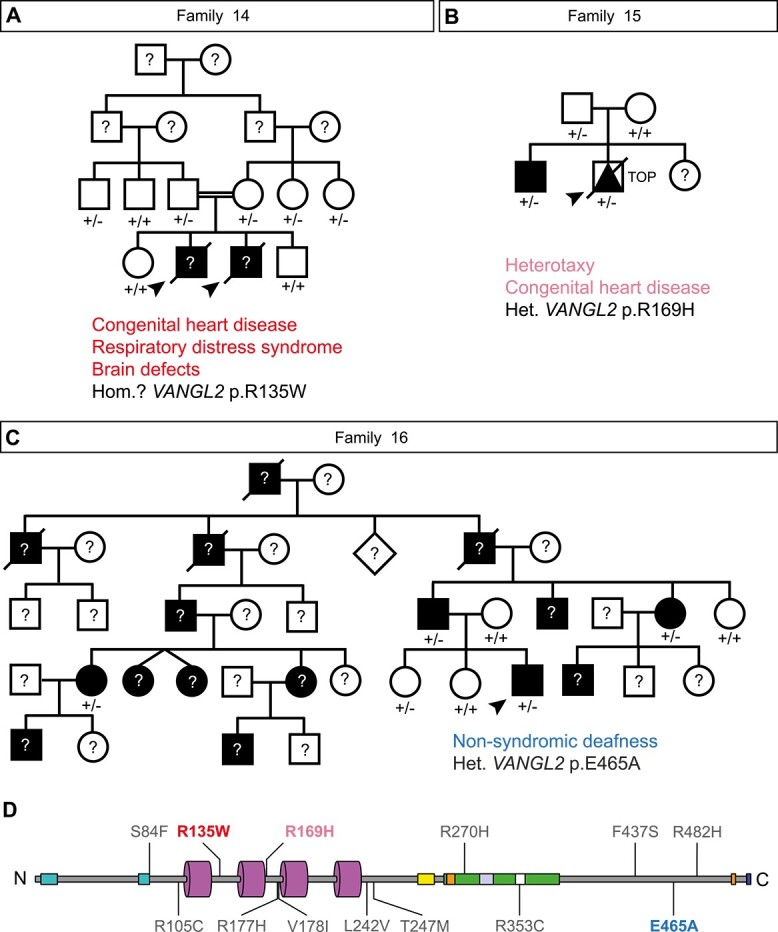
Identification of new *VANGL2* germline variants and families. (A) Clinical pedigrees of putative recessive p.R135W *VANGL2* family. Both deceased male probands (for which DNA was unavailable) possessed complete atrioventricular septal defect, respiratory distress syndrome, large occipital encephalocele and congenital hydrocephalus. Heterozygous family members were clinically normal. (B) Clinical pedigree of heterozygous p.R169H *VANGL2* family. Antenatal diagnosis of male proband identified dextrocardia, persistent left superior vena cava, total anomalous pulmonary venous drainage, atrioventricular septal defect, transposition of great arteries, pulmonary atresia, right pulmonary isomerism and asplenia. The elder brother is also heterozygous with situs inversus. The heterozygous father was clinically normal. (C) Clinical pedigree of heterozygous p.E465A *VANGL2* family in which proband has autosomal dominant non-syndromic hearing loss. The heterozygous p.E465A variant is also present in an unaffected relative. (D) Primary structure of Human VANGL2 showing known functional domains and location of previously reported and novel VUS reproduced from Fig. 2A. Squares, circles, diamonds, triangles represent male, females, unknown gender, and foetuses, respectively. Arrowheads denote proband. Diagonal line denotes deceased. Open symbols: healthy, black symbols: affected. Question mark denotes DNA unavailable. Het.: heterozygous, Hom: homozygous, TOP: termination of pregnancy.

The previously reported cases (Families 1–13) all exhibit a single proband. In contrast we have been referred three new families (Families 14–16, Table 2, Supplementary Table 1) where several members carry *VANGL2* missense alleles and display neurological defects, CHD, laterality disturbance or deafness (Fig. 3). These are all developmental abnormalities where a disruption to *VANGL2* function could be of relevance based on previous studies. In a consanguineous family (Family 14), two male neonates died within weeks of birth with atrioventricular septal defects (AVSD), respiratory distress syndrome, large occipital encephalocele and congenital hydrocephalus. Whilst DNA was not available from the affected infants, exome sequencing revealed both healthy parents to be heterozygous for *VANGL2* c.403C > T; p.(Arg135Trp), a variant already reported in a family with an unclear association with NTDs due to the presence of a heterozygous *DVL2* mutation (Fig. 2B Family 3, Fig. 3A). The remaining two healthy siblings did not carry the variant whilst three other family members carried the variant but were healthy. Since no other convincing variant was identified in the related parents, we hypothesized that the phenotype observed in the affected neonates might have been caused by homozygosity of this *VANGL2* variant. A novel *VANGL2* variant, c.506G > A; p.(Arg169His) was uncovered through exome sequencing of two male infants with laterality disturbance (Family 15, Fig. 3B). One child had complete situs inversus and one terminated male foetus exhibited heterotaxy, right isomerism and CHD. Sanger sequencing of the healthy parents revealed the father, like the children, to be a heterozygous carrier of this variant, yet the father was phenotypically normal. Finally, in a large family with dominant non-syndromic hearing loss (Family 16), exome sequencing identified a heterozygous *VANGL2* c.1394A> C; p.(Glu465Ala) variant in four affected individuals and one unaffected sibling (Fig. 3C). Again, similar to previously reported cases (Fig. 2), a clear, causal link between the origin of these malformations and the *VANGL2* VUS in sites which well conserved across evolution (Supplementary Fig. 1) is lacking and warrants further investigation.

### A zebrafish embryo functional assay for *VANGL2* variants

Throughout our study, we first began by defining an assay where injection of WT human *VANGL2* mRNA could at least partially rescue the zebrafish homozygous *vangl2* mutant phenotype. Here, we have used the *vangl2^m209^* allele, a known null [27] recovered from an ENU-mutagenesis screen [14, 64], in which retention of 13 bp of intronic sequence results in a frameshift and premature stop codon [15]. If WT human *VANGL2* mRNA was functional in *vangl2^m209^* rescue; we could then go on to examine whether human variant *VANGL2* mRNA could generate any form of rescue compared to the uninjected homozygous *vangl2^m209^* mutant and homozygous *vangl2^m209^* mutant injected with WT *VANGL2* mRNA. We explicitly chose to rescue using human *VANGL2* mRNA in order to control for species specific differences in the zebrafish *vangl2* gene that could obscure conclusions as to impact of the variant on gene function.

To begin, we defined a suitable dose of injected WT *VANGL2* mRNA that could rescue the CE defect of homozygous *vangl2^m209^* mutant zebrafish. The human *VANGL2* coding sequence (WT *VANGL2*) was cloned and a range of doses of WT *VANGL2* mRNA (70 pg to 280 pg) were injected at the 1-cell stage into homozygous *vangl2^m209^* mutants. The antero-posterior (AP) axis length, was measured at 24 h post fertilization (hpf) (Supplementary Fig. 2) as a readout of convergent-extension. Maximal, though not complete, rescue of AP axis length was achieved with 70–100 pg of WT *VANGL2* mRNA (Supplementary Fig. 2A–D and H), but amounts higher than 100 pg led to over-expression phenotypes, where injected mutants became indistinguishable from uninjected mutants (Supplementary Fig. 2E–H). Thus, 100 pg mRNA was used in all subsequent assays and is in keeping with previous reports for both *VANGL1* and *VANGL2* mRNA rescue [52, 77, 78]. Attempting to recapitulate the heterozygous state of human *VANGL2* VUS through injection of *VANGL2* variant mRNA into *vangl2^m209/+^* embryos was not possible as injection of WT *VANGL2* mRNA into *vangl2^m209/+^* siblings led to over-expression phenotypes, even at lower doses (Supplementary Fig. 3). Having confirmed the efficacy of WT *VANGL2* mRNA, we undertook site-directed mutagenesis of human WT *VANGL2* plasmid to introduce specific mutations of previously reported and novel *VANGL2* missense variants for functional *in vivo* testing by mRNA rescue.

### Most *VANGL2* variants retain convergent extension activity

**Figure 4:**
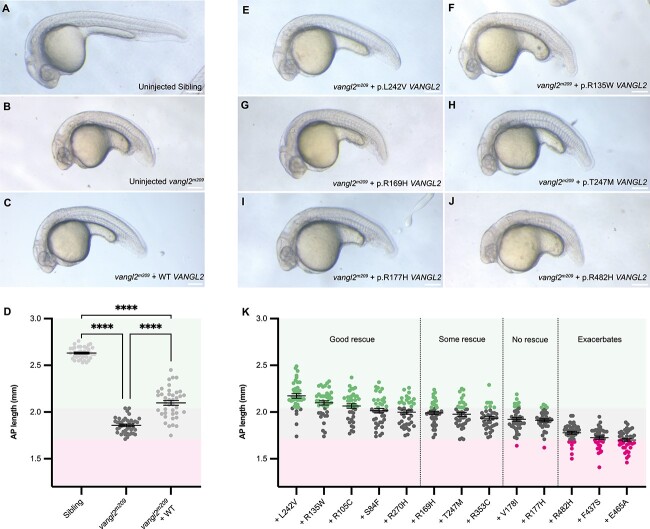
*vangl2^m209^* convergent extension defect can be rescued by most *VANGL2* VUS. (A–C) Representative brightfield images of (A) uninjected sibling, (B) uninjected homozygous *vangl2^m209^* mutant, (C) homozygous *vangl2^m209^* mutant injected with WT *VANGL2* mRNA. (D) Quantification of AP length at 26 hours post fertilization (hpf) to examine efficacy of WT *VANGL2* to rescue *vangl2^m209^* phenotype. Colour-coded regions defined by uninjected *vangl2^m209^* mutant data range: within/no rescue is grey, above/rescue is green and below/worsens is magenta. WT *VANGL2* mRNA injection partially rescues AP axis length. Each dot denotes a single embryo. (E) Homozygous *vangl2^m209^* mutant injected with p.L242V *VANGL2* mRNA, (F) homozygous *vangl2^m209^* mutant injected with p.R135W *VANGL2* mRNA, (G) homozygous *vangl2^m209^* mutant injected with p.R169H *VANGL2* mRNA, (H) homozygous *vangl2^m209^* mutant injected with p.T247M *VANGL2* mRNA, (I) homozygous *vangl2^m209^* mutant injected with p.R177H *VANGL2* mRNA, (J) homozygous *vangl2^m209^* mutant injected with p.R482H *VANGL2* mRNA. (K) Quantification of AP length at 26 hpf in homozygous *vangl2^m209^* mutants injected with mRNA encoding different *VANGL2* VUS. Variants are ranked and grouped based on result of statistical test when compared to uninjected homozygous *vangl2^m209^* mutants and homozygous *vangl2^m209^* mutants injected with WT *VANGL2* mRNA (see Supplementary Table 2 for statistical tests). Each dot denotes a single embryo. Colour coded regions and dots denote comparison against uninjected homozygous *vangl2^m209^* mutants from panel D. A-C, E-J: Lateral views, anterior left. D, K: Brown-Forsythe and Welch ANOVA with multiple comparisons, Mean ± SEM, ^*^^*^^*^^*^: p < 0.0001. Scale bars: 0.2 mm.

We first tested whether variants could rescue convergent extension, which is clinically relevant to the aetiology of NTDs and other lumbar-sacral malformations [64, 76, 77]. As shown in the initial titration experiments (Supplementary Fig. 2), injection of WT *VANGL2* into homozygous *vangl2^m209^* mutants was able to partially rescue CE (Fig. 4A–D). Five *VANGL2* variants were able to produce the same degree of rescue as WT *VANGL2* (p.Leu242Val), p.(Arg135Trp), p.(Arg105Cys), p.(Ser84Phe) and p.(Arg270His)), indicating a comparable degree of function to WT transcript (Fig. 4E–F and K, Supplementary Table 2). A lesser rescue was observed with p.(Arg169His), p.(Thr247Met), and p.(Arg353Cys) (Fig. 4G, H and K, Supplementary Table 2) whilst p.(Arg177His) and p.(Val178Ile) had no impact on AP axis length indicating no functional activity relating to convergent extension (Fig. 4I and K, Supplementary Table 2). Unexpectedly, injection of mRNA encoding the three variants localized at C-terminus (p.(Phe437Ser); (p.(Glu465Ala); and p.(Arg482His)) generated a significantly shorter AP axis length than observed in homozygous *vangl2^m209^* mutants (Fig. 4J and K, Supplementary Table 2, magenta region).

### Activity of *VANGL2* VUS in neuronal migration

Fundamental to the development of the nervous system is the migration of neurons to the location of their specialized function. In both mice and zebrafish, the migration of Islet1 (Isl1)-expressing facial branchiomotor (FBM) neurons requires PCP components which overlap with those required for neurulation and convergent extension [15, 16, 18–20]. To examine the impact of *VANGL2* VUS during FBM migration, we established a *vangl2^m209^; Tg(isl1a:GFP)* line [65] and imaged the extent of caudal migration of GFP-expressing FBM neurons from r4 to r6 at 36 hpf.

**Figure 5:**
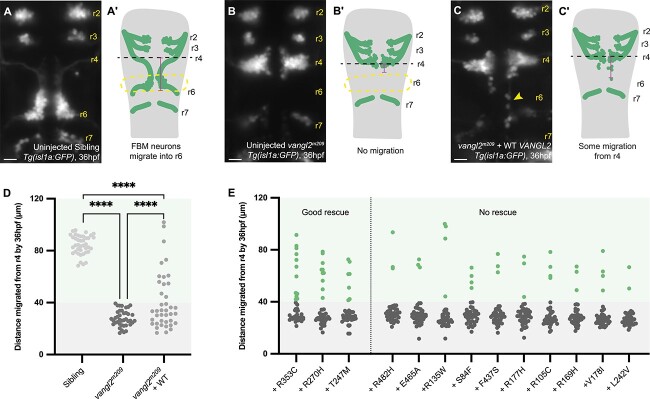
Motor neuron migration cannot be rescued by most *VANGL2* variants. (A–A′) Representative hindbrain of uninjected *Tg(isl1a:GFP)* sibling at 36 hpf (A), motor neurons have migrated from rhombomere 4 (r4) into rhombomere 6 (r6). Measurement of migration distance (magenta line) from r4 (black line) schematized in (A′). (B–B′) Representative hindbrain of *vangl2^m209^; Tg(isl1a:GFP)* homozygous mutant at 36 hpf (B), motor neurons have failed to migrate from r4 into r6. Measurement of migration distance schematized in (B′). (C–C′) Representative hindbrain of *vangl2^m209^; Tg(isl1a:GFP)* homozygous mutant at 36 hpf injected with WT *VANGL2* mRNA at 1-cell stage, some neurons exit r4 (arrowhead). Measurement of migration distance schematized in (C′). (D) Representation of Isl1a + neuron phenotype by migration distance from r4 at 36 hpf. Each dot denotes a single embryo. Colour-coded regions defined by uninjected homozygous *vangl2^m209^* mutant data range: within/no rescue is grey, above/rescue is green. In homozygous *vangl2^m209^* mutant embryos injected with WT *VANGL2* mRNA, migration distance above the mutant range is defined as “rescue”. p-values represent Fisher’s exact test based on rescue and mutant categories for each group. WT *VANGL2* mRNA injection partially rescues neuronal migration (see Supplementary Table 3 for statistical tests). (E) Representation of Isl1a + neuron phenotype by migration distance from r4 at 36 hpf in homozygous *vangl2^m209^* mutants injected with mRNA encoding different *VANGL2* VUS. Each dot denotes a single embryo, colour coded regions and dots denote comparison against the threshold of uninjected homozygous mutant data from panel D. Variants are ranked and grouped based on result of Fisher’s exact test when comparing rescue and mutant categories to uninjected homozygous *vangl2^m209^* mutants and homozygous *vangl2^m209^* mutants injected with WT *VANGL2* mRNA (see Supplementary Table 4 for statistical tests). A–C: dorsal views, anterior up. D-E: Fisher’s exact tests, ^*^^*^^*^^*^: p < 0.0001. Scale bars: 20 μm.

In wild-type siblings, the majority of FBM neurons migrated into r6 (Fig. 5A–A′), whilst in homozygous *vangl2^m209^* mutants, these cells completely failed to exit r4 (Fig. 5B–B′) [19]. By identifying neurons that were able to exit r4, a clear, stepwise phenotypic assay was developed (Fig. 5D). Injection of WT *VANGL2* mRNA into homozygous *vangl2^m209^* mutants led to an intermediate phenotype in some injected embryos, where some FBM neurons were observed to migrate out of r4 (Fig. 5C–C′ and D, green region, Supplementary Table 3). This demonstrates that similar to CE (Fig. 4), FBM migration can only be partially rescued by injection of WT *VANGL2* mRNA. To characterize the ability of *VANGL2* VUS to rescue FBM migration, we classified migration distance in an individual embryo injected with a given variant as ‘attempted migration’ (above the maximal distance migrated in uninjected *vangl2^m209^* mutants, (green region Fig. 5D and E) and ‘no migration’ (grey region Fig. 5D and E). Only three variants were able to produce comparable migration to WT mRNA: p.(Arg353Cys), p.(Arg270His) and (p.Thr274Met) (Fig. 5E, Supplementary Table 4).

### Neuromast polarity cannot be rescued by *VANGL2* mRNA injection

The elaboration of PCP is key for positioning and correct function of cilia within sensory structures [24]. Vangl2 has been shown to orient stereocilia in the mouse ear [25, 26] and analogous mechanosensory hair cells of the lateral line system in zebrafish [27]. During early development, otic stereocilia number is highly variable in zebrafish embryos [79] and are more challenging to image than the optically accessible neuromasts where the number of cilia is more consistent [80]. We therefore chose to examine impact of *VANGL2* VUS in stereocilia orientation in the lateral line system in view of the family exhibiting deafness with the p.(Glu465Ala) variant (Family 15, Fig. 3C).

The majority of cilia within the 1^st^ primI-derived neuromast (pL1) neuromast of wild type embryos were oriented along the AP axis but were randomized in homozygous *vangl2^m209^* mutants (Supplementary Fig. 4A–B′) [27]. Quantification confirmed this (Supplementary Fig. 4A″), with only 50% correctly orientated in *vangl2^m209^* null mutants (Supplementary Fig. 4B″). We observed no change in the orientation of stereocila when homozygous *vangl2^m209^* mutants were injected with WT *VANGL2* mRNA (Supplementary Fig. 4C–C″). We were therefore unable to establish a suitable assay for sensory cilia phenotypes.

### Disruption to asymmetric expression of *spaw* cannot be rescued by *VANGL2* patient variants

**Figure 6:**
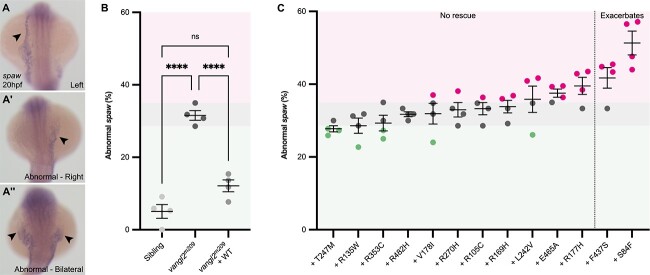
Asymmetric *spaw* expression in *vangl2^m209^* mutants cannot be rescued by *VANGL2* variants. (A–A″) Representative images of mRNA *in situ* hybridization for *southpaw* (*spaw*) the zebrafish homologue of *Nodal* at 20 hpf. In wild-type siblings (A), *spaw* is restricted to the left lateral plate mesoderm (LPM, arrowhead), alternatively, *spaw* may be abnormally expressed in the right LPM (A′, arrowhead) or bilaterally (A″, arrowheads). (B) Quantification of abnormal *spaw* expression at 20 hpf to examine efficacy of WT *VANGL2* to rescue homozygous *vangl2^m209^* mutant phenotype. WT *VANGL2* mRNA injection completely rescues *spaw* expression. Colour-coded regions defined by *vangl2^m209^* mutant data range: within/no rescue is grey, below/rescue is green and above/worsens is magenta. Each dot denotes a clutch, consisting of 17–30 embryos. (C) Quantification of abnormal *spaw* expression at 20 hpf in homozygous *vangl2^m209^* mutants injected with mRNA encoding different *VANGL2* VUS. Variants are ranked and grouped based on result of statistical test when compared to uninjected homozygous *vangl2^m209^* mutants and homozygous *vangl2^m209^* mutants injected with WT *VANGL2* mRNA (see Supplementary Table 5 for statistical tests). Each dot denotes a clutch, consisting of 18–43 embryos, colour coded regions and dots denote comparison against the uninjected homozygous *vangl2^m209^* mutant data from panel B. A–A″: Dorsal views, anterior up. B-C: One-way ANOVA with multiple comparisons, Mean ± SEM, ^*^^*^^*^^*^: p < 0.0001.

Vangl2 and PCP signalling have been strongly implicated in the establishment of the left–right axis during early embryogenesis [33, 34, 81–83] and the identification of a *VANGL2* VUS in a family with heterotaxy (Family 14, Fig. 3B) directed us to examine variant activity during left–right patterning. The zebrafish *Nodal* homologue *spaw* is one of the first asymmetrically restricted genes, normally expressed in the left LPM at 20 hpf (Fig. 6A). Right (Fig. 6A′), bilateral (Fig. 6A″) or absent *spaw* expression in the LPM (together defined as abnormal expression) was observed in approximately a third of homozygous *vangl2^m209^* mutant embryos and injection of WT *VANGL2* mRNA into homozygous *vangl2^m209^* mutants completely rescued this disrupted *spaw* expression (Fig. 6B). In contrast, none of the *VANGL2* human variants were able to rescue abnormal *spaw* expression in homozygous *vangl2^m209^* mutants (Fig. 6C, Supplementary Table 5). Notably, two variants, p.(Ser84Phe) and p.(Phe437Ser) (neither previously associated with heterotaxy), severely exacerbated the disruption to lateralized *spaw* expression, with almost 50% of *vangl2^m209^* mutant embryos injected with the p.(Ser84Phe) variant showing abnormal, particularly bilateral *spaw* expression (Fig. 6C, magenta region, Supplementary Fig. 5, Supplementary Table 5).

### Activity of *VANGL2* VUS during heart tube formation and positioning

Congenital malformations of the heart may be associated with disturbance of left–right patterning [84]. Separately, mice with mutations in *Vangl2* have highly penetrant heart malformations distinct from left-right patterning [23, 85], but genomic studies have not identified any association between *VANGL2* and CHD [5]. As one new proband in this study had heterotaxy with CHD (p.(Arg169His)) (Fig. 3B) and others possessed an AVSD, which may arise due to disruption to laterality [86] (p.(Arg135Trp)) (Fig. 3A), we specifically looked at early cardiac laterality in homozygous *vangl2^m209^* mutant zebrafish.

**Figure 7:**
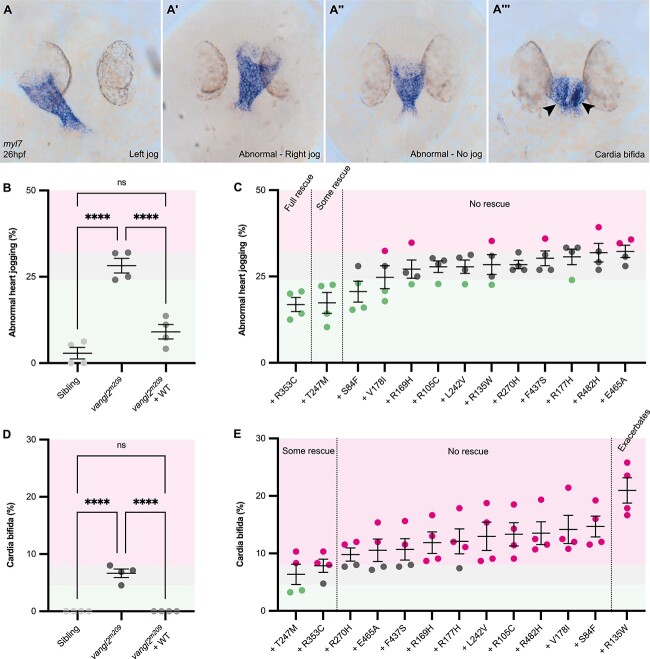
Heart tube formation and positioning is sensitive to loss of *vangl2* function. Representative heart tube phenotypes of homozygous *vangl2^m209^* mutant at 26 hpf, highlighted by mRNA *in situ* hybridization of the pan cardiac marker *myosin, light chain 7, regulatory* (*myl7*). (A) Normal, left jogging (A′) right jogging (A″) no jogging and (A‴) cardia bifida, the presence of two shorter heart tubes at the midline. (B) Quantification of abnormal heart jogging scored live between 28–30 hpf to examine efficacy of WT *VANGL2* to rescue homozygous *vangl2^m209^* mutant phenotype. WT *VANGL2* mRNA injection completely rescues heart jogging. Colour-coded regions defined by homozygous *vangl2^m209^* mutant data range: within/no rescue is grey, below/rescue is green and above/worsens is magenta. Each dot denotes a clutch, consisting of 16–29 embryos. (C) Quantification of abnormal heart jogging scored live between 28–30 hpf in homozygous *vangl2^m209^* mutants injected with mRNA encoding different *VANGL2* VUS. Variants are ranked and grouped based on result of statistical test when compared to uninjected homozygous *vangl2^m209^* mutants and homozygous *vangl2^m209^* mutants injected with WT *VANGL2* mRNA (see Supplementary Table 6 for statistical tests). Each dot denotes a clutch, consisting of 21–42 embryos, colour coded regions and dots denote comparison against the range of uninjected mutant data from panel B. (D) Quantification of cardia bifida scored live between 28–30hpf to examine efficacy of WT *VANGL2* to rescue homozygous *vangl2^m209^* phenotype. WT *VANGL2* mRNA injection completely rescues cardia bifida. Colour-coded regions defined by homozygous *vangl2^m209^* mutant data range: within/no rescue is grey, below/rescue is green and above/worsens is magenta. Each dot denotes a clutch, consisting of 16–29 embryos. (E) Quantification of cardia bifida scored live between 28–30 hpf in homozygous *vangl2^m209^* mutants injected with mRNA encoding different *VANGL2* VUS. Variants are ranked and grouped based on result of statistical test when compared to uninjected homozygous *vangl2^m209^* mutants and homozygous *vangl2^m209^* mutants injected with WT *VANGL2* mRNA (see Supplementary Table 7 for statistical tests). Each dot denotes a clutch, consisting of 21–42 embryos, colour coded regions and dots denote comparison against uninjected homozygous *vangl2^m209^* mutant data from panel D. Heart jogging and cardia bifida were analysed in the same clutch. A–A‴: dorsal views, anterior up. B-E: One-way ANOVA with multiple comparisons, Mean ± SEM, ^*^^*^^*^^*^: p < 0.0001.

The zebrafish linear heart tube is normally positioned under the left eye by a process known as jogging [74, 87] (Fig. 7A). We identified four different heart phenotypes in homozygous *vangl2^m209^* mutants at 26 hpf: normal left jog (Fig. 7A), right jog (Fig. 7A′), no jog (midline heart, Fig. 7A″) and a delayed fusion of heart fields leading to cardia bifida (two shorter heart tubes at the midline) (Fig. 7A‴). Abnormal jogging (right jog or no jog) was present in around 30% of homozygous *vangl2^m209^* mutants (Fig. 7B). Cardia bifida was completely absent in wild-type siblings and present in approximately 6% of homozygous *vangl2^m209^* mutants (Fig. 7D).

Injection of WT *VANGL2* mRNA into homozygous *vangl2^m209^* mutants completely rescued both disruption to heart jogging (Fig. 7B) and cardia bifida (Fig. 7D). *VANGL2* p.(Arg353Cys) mRNA was capable of rescuing abnormal heart jogging to the same extent as WT transcript, suggesting a full rescue, but could only partly rescue cardia bifida (Fig. 7C and E, Supplementary Tables 6 and 7). p.(Thr247Met) *VANGL2* mRNA could partially rescue both abnormal heart jogging and heart tube formation (Fig. 7C and E, Supplementary Tables 6 and 7). None of the other 11 transcripts were capable of rescuing either abnormal jogging or cardia bifida (Fig. 7C and E, green region, Supplementary Fig. 6, Supplementary Table 5), however p.(Arg135Trp) *VANGL2* greatly increased the incidence of cardia bifida (Fig. 7E, magenta region, Supplementary Fig. 6, Supplementary Table 7).

### Variant *in vivo* function does not correlate with *in silico* prediction

**Figure 8:**
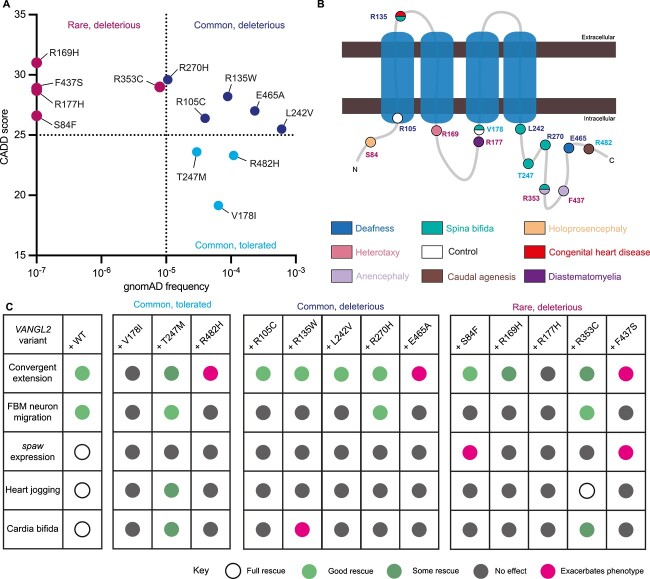
Summary of *VANGL2* VUS *in vivo* activity. (A) Comparison of observed variant frequency and CADD score of *VANGL2* variants. p.S84F, p.R169H, p.R177H and p.F437S have not been observed in the population and are assigned frequency of 10^−7^. (B) Schematic of VANGL2 protein and location of residues, amino acid (text) is colour coded based on (A), residue position is colour coded based on malformation associated with variant (Figs 2 and 3). (C) Summary table of *vangl2*-developmental processes that were able to be at least partially rescued by injection of WT *VANGL2* mRNA and results of variant mRNA analysis. Full rescue denoted by open circles, good rescue: green, some rescue: dark green, no rescue: grey, and exacerbation: magenta.

Use of predictive *in silico* tools of variant impact, despite only 65%–80% accuracy [45] is routine for ascribing pathogenicity to variants linked to Mendelian disorders [44]. Whilst it is unclear whether *VANGL2* mutations and associated disease phenotypes follow Mendelian genetics, we chose to examine whether this approach could complement *in vivo* functional testing. We grouped variants based on gnomAD frequency and CADD score (Tables 1, 2, Supplementary Table 1, Fig. 8A) in an attempt to highlight variants potentially causative of the associated disease [44]. From this, we identified three categories: rare and predicted to be deleterious (p.(Ser84Phe), p.(Arg177His), p.(Arg169His), p.(Arg353Cys) and p.(Phe437Ser)); common and predicted to be deleterious (p.(Arg105Cys), p.Arg135Trp), p.(Leu242Val), p.(Arg270His) and p.(Glu465Ala)); and common and predicted to be tolerated p.(Val178Ile), p.(Thr247Met) and p.(Arg482His)) (Fig. 8A).

We found no strong relationship between prediction (Fig. 8A), position on VANGL2 peptide, (Fig. 8B) nor transcript’s rescue capability in our assays (Fig. 8C). Importantly, although most deleterious variants (with the exception of p.(Arg353Cys)) failed to rescue in the majority of our assays, distinct developmental processes were impacted differently by the same variant (Fig. 8C). This suggests *in silico* predictions alone may miss more nuanced, organ-specific impacts of a given variant. We also noted when correlating patient phenotype with variant position on the VANGL2 peptide (Fig. 8B) some suggestion that variants in the C-terminal domain may be more associated with NTDs, whilst more N-terminal variants relate to laterality disturbances, with or without CHD. Yet with the limited number of cases and phenotypic information available, it is not possible to be certain about this, but this concept may be relevant for future studies in other genes.

## Discussion

With the advent of rapid DNA sequencing, the assignment of gene variants to clinical presentations has become commonplace. However, many of these variants are of unknown or unproven significance with no functional *in vivo* tests to establish a causative link between specific mutations and patient phenotypes. In this study, we developed and carried out a series of functionally relevant analyses of both novel and previously reported *VANGL2* VUS using mRNA rescue of the *vangl2^m209^* null zebrafish. Although injection of variant *VANGL2* mRNA into *vangl2^m209/+^* embryos may more closely mimic the heterozygous state of the probands, this was not possible as injection of WT *VANGL2* mRNA in *vangl2^m209/+^* embryos caused significant CE defects (Supplementary Fig. 3). Furthermore, it is unclear what the appropriate dosage would be to achieve endogenous levels and this may lead to weaker phenotypes that would have been more challenging to interpret in an initial screen. Thus, our pipeline has allowed us to assess the functionality of variants in specific developmental scenarios, rather than simulating the genetic composition of the probands. This has enabled a simplified approach that can be built upon by further studies. Here, we have interpreted our functional readouts and where sufficient clinical and genetic data exists, we make suggestions as to the potentially pathogenic role of these *VANGL2* variants in the associated human disease.

A clear limitation of mRNA injection approaches is the lack of transcriptional regulation and loss of potential splice variants. Although this could be overcome by making knock-in mutants for each variant, this is time consuming and expensive. A specific advantage of using human *VANGL2* transcript to rescue the homozygous *vangl2^m209^* phenotype is that it allows conclusions to be drawn about human variants that are not conserved in zebrafish (Supplementary Fig. 1). For example, *VANGL2* mRNA containing the non-conserved c532G > A p.(Val178Ile) variant (Supplementary Fig. 1E), was unable to rescue any of the developmental processes we assessed (Fig. 8C). It is possible that the heterozygous p.(Val178Ile) mutation may have variable penetrance of phenotype, leading to malformation in some individuals, whilst others are unaffected (Fig. 2, Families 5 and 6). Much more likely, given the high prevalence of heterozygous p.(Val178Ile) in the population (Table 1, Fig. 8A) and the absence of clinical phenotype in almost all cases of p.(Val178Ile), is that this variant is not pathogenic when heterozygous and another genetic anomaly underlies tethered cord in the patient, which was not identified due to the targeted sequencing approach of the original study. However, our assays do suggest that when homozygous, the p.(Val178Ile) variant would be pathogenic as it was unable to rescue any *vangl2*-dependent process that we assayed (Fig. 8C). This is in contrast to the *in silico* analysis, which defined this amino acid substitution as benign (Table 1, Fig. 8A). The neighbouring c530G > A p.(Arg177His) variant was identified in a proband with diastematomyelia (split spinal cord), was predicted to be deleterious, and also showed no rescue capability in any assay (Fig. 8C). We suggest this variant is likely to be pathogenic, although the absence of other, more severe malformations could suggest genetic modifiers functioning in this family [88]. In contrast, the c740C > T, p.(Thr247Met) variant, which affects a non-conserved residue between zebrafish and human (Supplementary Fig. 1F) was able to function similarly to WT *VANGL2* in all the assays except rescue of *spaw* expression (Fig. 8C), thus it does not seem capable of causing meningomyelocele and is likely to be a benign variant in this developmental context, as supported by *in silico* prediction (Fig. 8A). Together this demonstrates the need for full genome sequencing of patients and families, as well as *in vivo* testing of variant activities, as predictive *in silico* tools may not always be accurate.

Development of sensory cilia in both the zebrafish ear and lateral line are well understood however, we could not establish an assay using mRNA rescue to evaluate particularly the c.1394A > C; p.(Glu465Ala) variant, which appears to segregate with non-syndromic deafness (Family 16). However, this variant is deleterious as it failed to function in the other assays and significantly worsened CE (Fig. 8C). The pedigree indicates at least one family member with normal hearing also carries the variant (Fig. 3C), suggesting possible partial penetrance or linkage with another regulatory element or gene which is functionally responsible for deafness. Therefore, this variant remains a variant of unknown significance.

Heart malformations are a feature of *Vangl2* mutant mice, but the zebrafish *vangl2* cardiac phenotype has been relatively poorly described. Here we have shown that in homozygous *vangl2^m209^* mutants, there is both abnormal migration of first heart field cardiac progenitors leading to two heart tubes (cardia bifida) as well as abnormal heart tube positioning. Examining early tube formation and positioning was the principle assay for family 14 (Fig. 7), where the probands presented with congenital heart malformation (Fig. 3B). This c.403C > T; p.(Arg135Trp) variant increased the frequency of cardia bifida in homozygous mutants and was unable to rescue the cardiac jogging, neural migration or *spaw* expression phenotypes (Fig. 8C). Thus our data supports the idea that this variant could be related to the CHD observed in the patients.

Due to absence of DNA for the affected siblings of family 14, whole exome sequencing was performed on both parents, which identified the heterozygous *VANGL2* p.(Arg135Trp) variant in both individuals and other healthy family members (Fig. 3A). Since no other convincing variant was identified in the related parents, we speculate that the phenotype observed in the affected neonates may have been caused by homozygosity of this *VANGL2* variant. Interestingly, if so, there are clear differences between the hypothetically recessive p.(Arg135Trp) *VANGL2* patient malformations and both neural and cardiovascular defects observed in mouse mutants for *Vangl2*. Firstly, *Vangl2* mutant mice develop ventricular septal defects (VSD) [23, 85], but not AVSD which we report in both neonates. One explanation may relate to the link between AVSD and laterality disruption [86] or that the presence of AVSD may mask VSD; this warrants further investigation. The second difference between the putative p.(Arg135Trp) homozygous patients and *Vangl2* mutant mice is the absence of any NTD in the probands, particularly craniorachischisis which is fully penetrant in *Lp* mutants due to failure of CE [22]. We have shown that p.(Arg135Trp) *VANGL2* mRNA is able to rescue CE similarly to WT transcript (Figs 4F, K and 8C and Supplementary Table 1), potentially explaining the absence of a NTD in these patients. Finally, as downstream effectors of PCP signalling during heart tube formation are distinct from those required for CE [89, 90] this may explain the differences between rescue of CE and cardiac phenotypes.

Our *in vivo* analysis may also explain the presence of the NTD in the historic case of p.(Arg135Trp) that was also carrying a heterozygous *DVL2* mutation (Fig. 2, Family 3). In our new p.(Arg135Trp) case (Fig. 3A), there were no NTDs reported in the heterozygous carriers, therefore it seems likely that the NTD in the p.(Arg135Trp) *VANGL2*; p.(Thr535Ile) *DVL2* case is due to the genetic interaction between these two mutations. This is well supported in the literature as digenic heterozygous mice for *Vangl2* and other PCP genes develop severe NTDs, whilst the single heterozygotes develop normally [91]. In the other case of a *VANGL2*; *DVL2* heterozygous proband (family 13), the p.(Arg482His) *VANGL2* variant is clearly deleterious as it was unable to rescue any *vangl2^m209^* phenotypes and exacerbated CE (Fig. 8C). However, whether the *VANGL2* mutation alone would be sufficient to result in caudal agenesis is unclear.

Heterotaxy, the combination of cardiac malformation and left–right patterning defects, was the principle phenotype of Family 15 and the role of the c.506G > A; p.(Arg169His) variant was assayed through examining *spaw* expression (Fig. 6). This variant was not able to rescue any developmental phenotype except for CE and as such is likely to be a pathological variant. The different phenotypes observed within Family 15: an asymptomatic father, situs inversus in the first sibling and heterotaxy in the second, are not unexpected given that disruption of left–right patterning is known to lead to randomization of organ placement resulting in variability of phenotype, including normal placement by chance. Together, this suggests that a single copy of p.(Arg135Trp) is unlikely to be sufficient to disrupt left–right patterning (as no heterotaxy is reported in heterozygous carriers in either family 3 or 15), but a single copy of p.(Arg169His) *VANGL2* may be haploinsufficient and cause heterotaxy. Importantly, these conclusions suggest that the site of a *VANGL2* variant as well as copy number may have different impacts on development (Fig. 8B).

The proband in family 1 (p.(Ser84Phe)) had holoprosencephaly, the failure of embryonic forebrain division, which has been associated with disruption to left–right patterning [91, 92]. Supporting a distinct aetiology of holoprosencephaly from NTDs there was preservation of CE activity comparable with WT transcript (Fig. 4K, Supplementary Table 1), although the variant was not able to rescue in other assays (Fig. 8C). Most strikingly, the variant significantly increased abnormal bilateral *spaw* expression (Supplementary Fig. 5), which is in keeping with abnormalities in midline specification. The Ser84 residue of Vangl2 is located in one of the two N-terminal clusters of Serine/Threonine residues (Figs 2A and 3D) that are phosphorylated in response to Wnt5a to position nodal cilia and establish the left–right axis [78, 93]. Furthermore, with a clinical link between *NODAL* and holoprosencephaly [91, 92, 94], our results support that p.(Ser84Phe) is a pathological variant.

The p.(Phe437Ser) variant (Family 12) also showed significantly increased abnormal *spaw* expression, although the exact clinical relevance of this is unclear (Fig. 8C). This variant has been linked with anencephaly, a failure in primary neurulation [95]. The exacerbation of the *vangl2* CE phenotype by p.(Phe437Ser) *VANGL2* mRNA in zebrafish could support a pathogenic role of this variant.

The c.1057C > T p.(Arg353Cys) variant in Family 11 was identified in a foetus with anencephaly and spina bifida. However, this variant was capable of rescuing all *vangl2^m209^* phenotypes except *spaw* expression (Fig. 8C). If this variant were pathogenic, it would be expected to have failed to rescue CE, suggesting it is unlikely to be singularly causative. Similarly, the p.(Arg270His) variant in Family 10 in a proband with fibrolipoma of filum terminus produced good rescue for CE and neuronal migration (Fig. 8C), which may suggest another cause for the patient phenotype.

Interestingly, some of the variants assessed in this study worsened the *vangl2^m209^* phenotype, although these roles seem context dependent (Fig. 8C). This may be due to active rather than passive interference with maternally deposited zygotic *vangl2* transcript and may indicate a higher level of variant pathogenicity. The three variants which exacerbated the *vangl2^m209^* CE phenotype (p.(Phe437Ser), p.(Glu465Ala) and p.(Arg482His)) reside in the C-terminal domain of Vangl2 (Fig. 8B), known to interact with Dvl [96] a master regulator of Wnt signalling [97]. p.(Phe437Ser) abolishes Dvl interactions [75], and similarly p.(Glu465Ala) is next to Ser464, which when mutated in mice, prevents Dvl interactions in culture [75, 98]. One molecular mechanism may be a failure of Vangl2-Dvl binding that is necessary to inhibit Dvl-Daam1 interactions during convergent extension [99]. What role Arg482 plays is unknown, but the similarity in CE rescue may suggest it also plays a role in Dvl interactions.

The exacerbation of *spaw* by p.(Ser84Phe) *VANGL2* may be linked to the role of the Ser/Thr cluster in node polarization [78], yet the basis of a similar phenotype in *vangl2^m209^* mutants injected with p.(Phe437Ser) *VANGL2* mRNA is less clear. Both Wnt/β-catenin and Wnt/PCP-signalling are required for left–right specification [100] and both pathways utilize Dvl [97, 101], with which the p.(Phe437Ser) variant cannot interact [75]. Vangl2 can repress Wnt/β-catenin signalling [102, 103] and coupled with the failure of the p.(Phe437Ser) Vangl2 to interact with Dvl, this could lead to an altered balance between Wnt signalling pathways, with increased Wnt/β-catenin signalling leading to higher penetrance of bilateral *spaw*, a similar phenotype observed upon loss of Nkd1, which regulates Dvl [104].

p.(Arg135Trp), the only variant identified for *VANGL2* that is present on the extracellular surface (Fig. 8B), worsens the cardia bifida phenotype (Fig. 8C). Loss of the extracellular matrix protein fibronectin also results in cardia bifida [105, 106], therefore it could be that this variant impacts extracellular interactions that are necessary for cardiomyocyte migration. However, there appears to be a complex, reciprocal interaction between Vangl2 and fbronectin as a fibronectin containing matrix is required for membrane localization of Vangl2 during gastrulation [107] but *vangl2* is required fibronectin assembly [108]. This could make untangling the nature of failure and possible worsening of cardia bifida challenging.

Despite having evaluated these variants and made suggestions regarding their pathogenicity, significant uncertainty remains and there is no basis for prospective genetic counselling for families or individuals with *VANGL2* variants. In part, this is due to insufficient clinical and genetic information provided in the majority of these probands and their families where other background mutations are likely to be relevant [88, 109]. Especially for historic cases where targeted sequencing rather than unbiased approaches were undertaken, it is hard to be definitive about a specific variant without knowledge of the complete genome. This could also explain perceived variable penetrance of phenotypes and identification of variants in control populations. A further, important consideration is that it may be more accurate to initially assume a non-Mendelian mode of function for *VANGL2* variants given that copy number and site of mutation also appears to impact pathogenicity. Moreover, pedigree information and sporadic cases do not confirm such classical inheritance patterns.

Our pipelines examined the rescue ability of a given variant, rather than recreating the exact patient genetics. With this caveat, the majority of the variants we tested had the potential to cause cardiac malformation, although this was not noted in pedigrees of clinical family histories. Furthermore, *VANGL2* variants do not appear to be a common feature in foetuses or infants with CNS malformations or hearing loss. There are several possible explanations for this. Firstly, it may be that these transcripts are genuine pathological variants with high penetrance, but in human populations they are generally lethal at an early stage of development and hence the phenotypes of patients reported as less severe as these individuals represent survivors of development. As noted above, traditional Mendelian concepts may not accurately represent *VANGL2* function, since variants appear to act variably in the heterozygote state and we have no explanation as to why such variants should have such profound effects. Alternatively, as almost all of these variants were identified in heterozygous patients (Figs 2 and 3), dimerization with wild type VANGL2 or VANGL1 may impair normal pathway activity [110, 111] with variable penetrance; a notion supported by the low incidence of spina bifida in heterozygous *Vangl2* mutant mice [22]. Finally, it may be that these variants are not actually causative, but are indicative of abnormalities in the genetic landscape that surrounds the *VANGL2* gene. These possibilities are in keeping with a meta-analysis demonstrating no direct association between the historic *VANGL2* variants (Fig. 2) and detectable NTD risk [38].

In conclusion, after completing these studies, it is clear that many complementary tracks of information are required to understand the contribution of genetic variation to human developmental abnormalities. Firstly, accurate, unbiased, phenotypic characterization of probands and other family members spanning over several generations is required to identify syndromic and familial inheritance patterns. Secondly, in depth genetic information is required to identify variants in relevant and accessory developmental pathways as well as understanding the genetic background of patients. As a basic threshold this needs to be next generation whole exome sequencing, but as the complexity of non-exomic chromatin begins to be understood, whole genome sequencing will be important, particularly when considering genetic modifiers [88, 109]. Thus, a full appreciation of the genome is relevant, especially with the identification of individuals termed “superheroes of disease resistance” which could be considered an extreme example of incomplete penetrace [111–113]. In these rare cases, individuals harbouring either heterozygous or homozygous missense variants in genes known to cause diseases in humans have no clinical symptoms [114]. This suggests that the ability of an individual to develop and survive, despite potentially deleterious genetic variability (genetic robustness) [115] is an important consideration when defining a single mutation in a non-syndromic gene as causative. Thirdly, there is a need to understand the development of the relevant organ system in order to ascribe plausible biological relevance to the variants. Fourthly, there needs to be a meaningful assay that can objectively validate the potential of a variant to be disruptive without an over-reliance on *in silico* prediction tools particularly as we have shown that variants may have organ-specific impacts (Fig. 8C). However, these computational analyses should be taken in to consideration, particularly in relation to frequency of variants in a population. Finally, as almost all variants are found in the heterozygote state and the vast majority of cases do not follow classical Mendelian genetics, there needs to be critical and objective thinking about whether these variants are directly causative or whether they reflect other non-classical genetic disturbances that instead facilitate malformation.

## Supplementary Material

HMG_2023_CE_00479_Derrick_Szenker_Ravi_Supplemental_ddad171

## Data Availability

All data that supports the findings not presented in main figures and supplementary files are available from the relevant corresponding author upon request.
