# Supplementary material for: Functional analysis of germline VANGL2 variants using rescue assays of vangl2 knockout zebrafish
Source: Hum Mol Genet. 2023 Oct 10;33(2):150–69. doi: 10.1093/hmg/ddad171 (PMC10772043; doi:10.1093/hmg/ddad171)
Supplement: HMG_2023_CE_00479_Derrick_Szenker_Ravi_Supplemental_ddad171 [file hmg_2023_ce_00479_derrick_szenker_ravi_supplemental_ddad171.pdf]

Functional analysis of germline *VANGL2* variants using rescue assays of *vangl2* knockout zebrafish

Derrick, Szenker-Ravi et al.

Authors for correspondence:

Bruno Reversade

Smart-Health Initiative, BESE, KAUST, Thuwal, Kingdom of Saudi Arabia

Telephone: (+65) 6407 0169

Email: bruno@reversade.com

ORCiD: 0000-0002-4070-7997

Bill Chaudhry

Biosciences Institute, Newcastle University, International Centre for Life, Central Parkway, NE1 3BZ, United Kingdom

Telephone: +44(0)191 241 8681

Email: bill.chaudhry@ncl.ac.uk

ORCiD: 0000-0003-2833-8882

Supplemental Material:

Figures S1-6

Tables S1-8

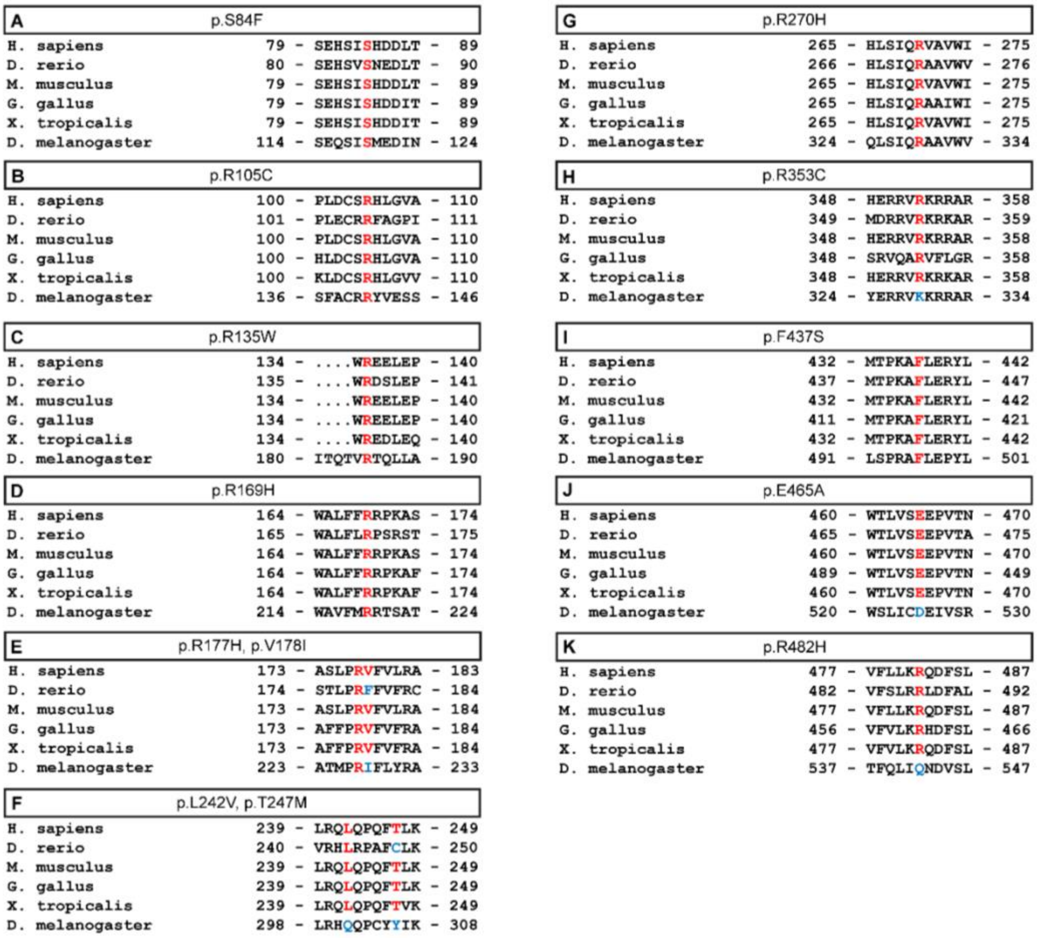

Supplemental Figure 1. Conservation of VANGL2 residues mutated in patients

(A-K) Sequence comparisons of residues affected in *VANGL2* VUS, aligned by Clustal Omega with zebrafish (UniProt: Q8UVJ6), mouse (UniProt: Q91ZD4), chick (UniProt: A0A1D5PV80), *Xenopus tropicalis* (UniProt: Q4VBG5) and the originally identified *Drosophila* homolog Strabismus (UniProt: A1Z7N9). Residues conserved with Human VANGL2 are highlighted in red, non-conserved residues in blue.

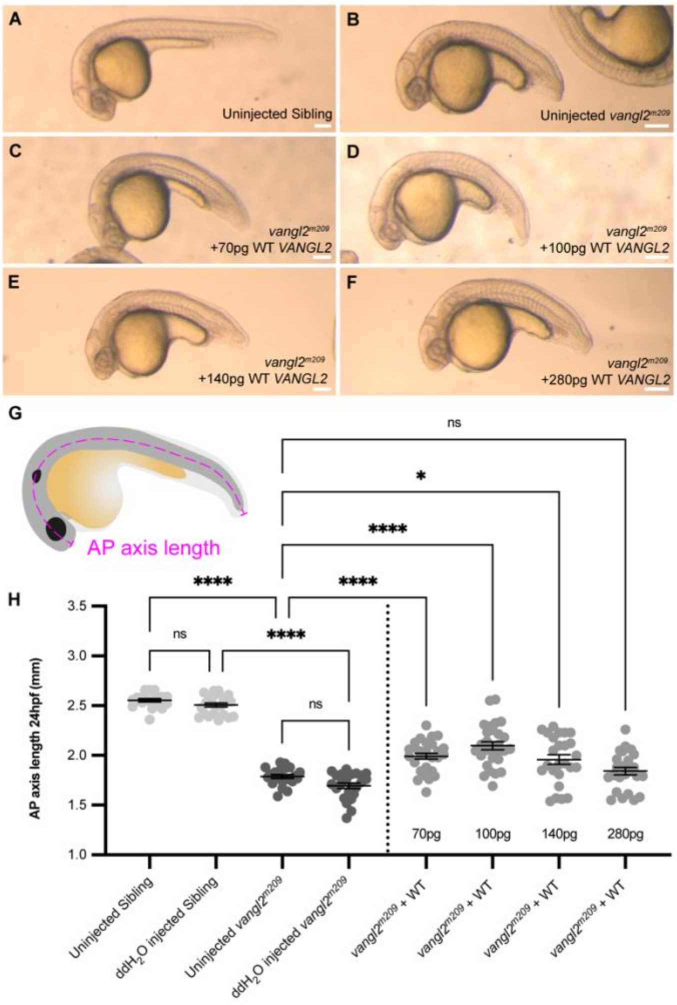

Supplemental Figure 2. Titration of optimal concentration of WT *VANGL2* mRNA for rescue for VUS testing

(A-F) Representative brightfield images of (A) uninjected sibling, (B) uninjected *vangl2*^{m209} homozygous mutant, (C) *vangl2*^{m209} homozygous mutant injected with 70pg WT *VANGL2* mRNA, (D) *vangl2*^{m209} homozygous mutant injected with 100pg WT *VANGL2* mRNA, (E) *vangl2*^{m209} homozygous mutant injected with 140pg WT *VANGL2* mRNA, (F) *vangl2*^{m209} homozygous mutant injected with 280pg WT *VANGL2* mRNA. **(G)** Schematic demonstrating how antero-posterior (AP) length is measured. **(H)** Quantification of AP length at 24 hours post fertilisation (hpf) to examine efficacy of different doses of WT *VANGL2* to rescue *vangl2*^{m209} homozygous mutant phenotype. Each dot denotes a single embryo (n=20-29 per condition). Mock injection of ddH_2O has no impact. WT *VANGL2*

mRNA injection is able to partially rescue AP axis length optimally between 70-100pg. A-F: lateral views, anterior left. H: Brown-Forsythe and Welch ANOVA with multiple comparisons, Mean ± SEM, ns: not significant, *: $p<0.05$, ****: $p<0.0001$. Scale bars: 0.2mm

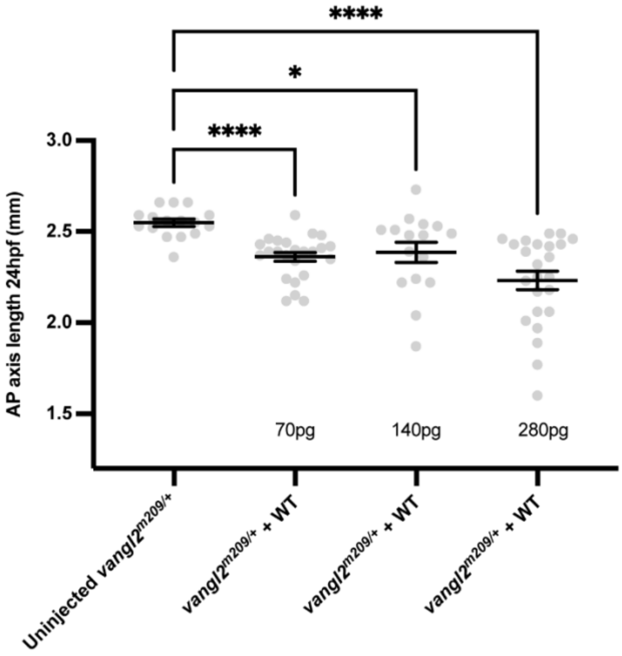

Supplemental Figure 3. Injection of WT *VANGL2* mRNA into *vangl2$^{m209/+}$* results in over-expression phenotypes

Quantification of AP length at 24 hours post fertilisation (hpf) in *vangl2$^{m209/+}$* embryos injected with a range of WT *VANGL2* mRNA. Each dot denotes a single embryo (n=20-24 per condition). Injection of WT *VANGL2* mRNA causes significant reduction in AP axis length. H: Brown-Forsythe and Welch ANOVA with multiple comparisons, Mean ± SEM, *: p<0.01, ****: p<0.0001.

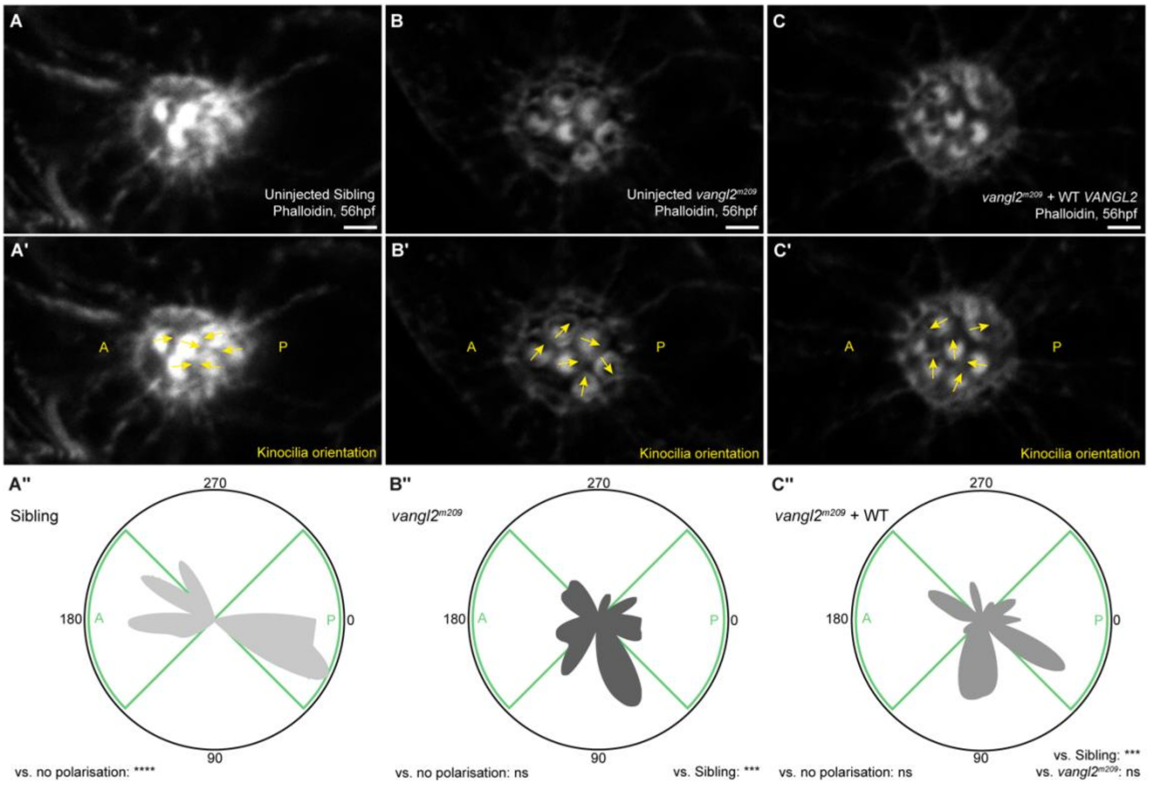

Supplemental Figure 4. *VANGL2* mRNA injection cannot rescue *vangl2^{m209}* pL1 kinocilia polarity

(A-A') Representative left pL1 from uninjected sibling at 56 hpf stained with Phalloidin identifying cell orientation (A', arrows) relative to AP axis. **(A'')** Rose plot displaying percentage of individual hair cells aligned relative to horizontal axis of in uninjected siblings (n=8). Uninjected siblings primI-derived pL1 neuromast displays a strong planar polarisation of hair cells along the AP axis of the embryo. Testing AP (86%, 37/43, green quadrants) and DV (14%, 6/43) alignment against no polarisation (AP 50%, DV 50%), confirms a significant polarisation. **(B-B')** Representative left pL1 from uninjected homozygous *vangl2^{m209}* mutant at 56 hpf stained with Phalloidin identifying cell orientation (B', arrows) relative to AP axis (n=8). **(B'')** Rose plot displaying percentage of individual hair cells aligned relative to horizontal axis of Uninjected homozygous *vangl2^{m209}* mutants. Loss of *vangl2* results in randomisation of hair cell orientation in left pL1 neuromast. Testing AP

(50%, 20/40 green quadrants) and DV (50%, 20/40) alignment in Uninjected homozygous *vangl2*^{m209} mutants against no polarisation (AP 50%, DV 50%), confirms no polarisation. Fisher's exact test comparing orientations of cilia in AP and DV orientations in uninjected homozygous *vangl2*^{m209} mutant to siblings shows a significant difference. **(C-C')** Representative left pL1 at 56 hpf from an homozygous *vangl2*^{m209} mutant injected with WT *VANGL2* mRNA, stained with Phalloidin to identify cell orientation (C', arrows) relative to AP axis (n=8). **(C'')** Rose plot displaying percentage of individual hair cells aligned relative to horizontal axis of homozygous *vangl2*^{m209} mutants injected with WT *VANGL2*. Injection of WT *VANGL2* does not rescue hair cell orientation in homozygous *vangl2*^{m209} mutants. Testing AP (50%, 19/38, green quadrants) and DV (50%, 19/38) alignment in homozygous *vangl2*^{m209} mutants injected with WT *VANGL2* mRNA against no polarisation (AP 50%, DV 50%), confirms no polarisation. Fisher's exact test comparing orientations of cilia in AP and DV orientations in homozygous *vangl2*^{m209} mutants injected with WT *VANGL2* to siblings shows a significant difference, whilst comparisons with uninjected homozygous *vangl2*^{m209} mutants shows no significant difference. Siblings are uninjected controls from same cross as uninjected mutants. A-C': lateral views, anterior left, scale bars: 2µm. A'': two-tailed binomial test, B'', C'': two-tailed binomial test for tests for polarisation, Fisher's exact test when comparing experimental groups. Rose plots represent summary of individual cilia (4-6 per embryo), pooled from 8 separate embryos. ns; not significant, ***: p<0.001, ****: p<0.0001. A: Anterior, P: Posterior.

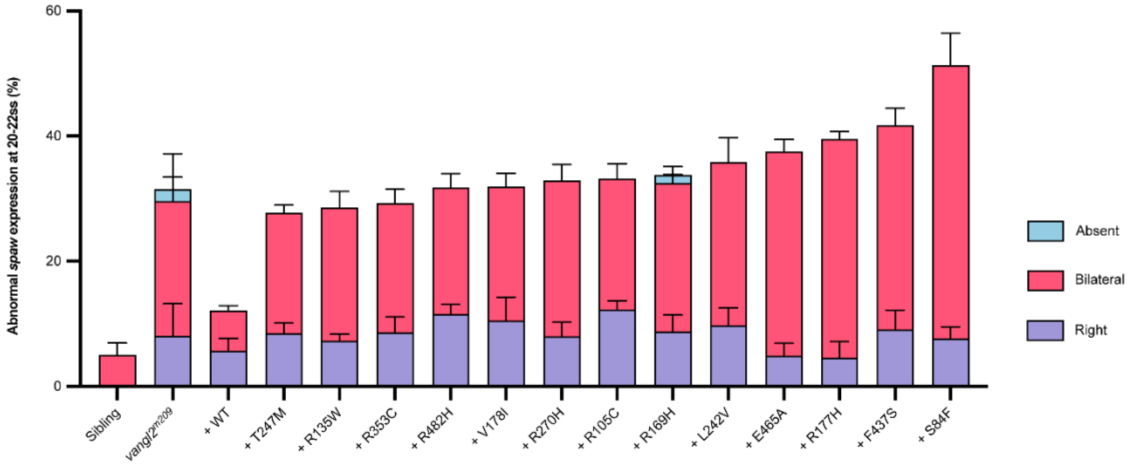

Supplemental Figure 5. Analysis of *spaw* expression in *vangl2*^{m209} mutants injected with *VANGL2* VUS mRNA

Quantification of abnormal *spaw* expression at 20 hpf in all experimental cases from Figure 6 separated into right, and bilateral and absent. Siblings are uninjected controls from same cross as uninjected mutants. N=4, Mean ± SEM.

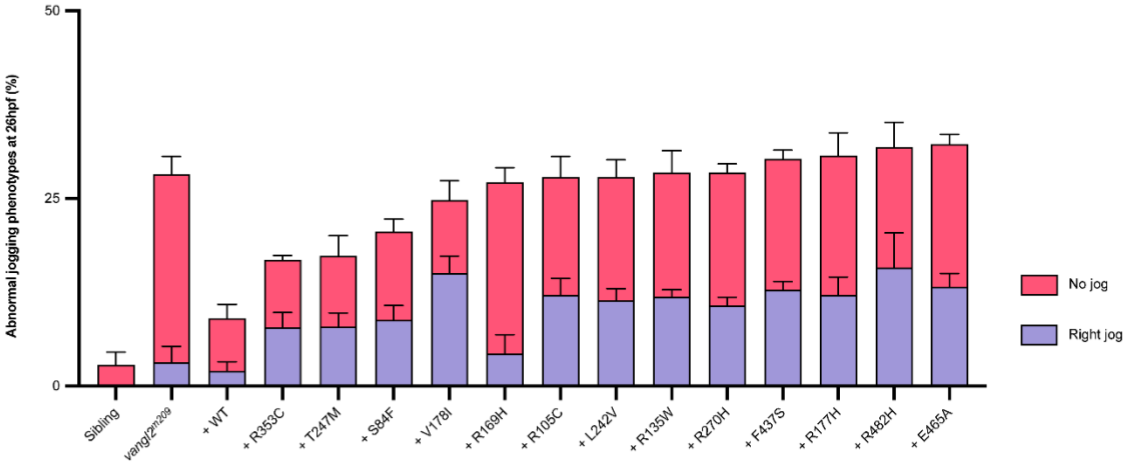

Supplemental Figure 6. Analysis of jogging direction in *vangl2^{m209}* mutants injected with *VANGL2* VUS mRNA

Quantification of abnormal jogging in all experimental cases from Figure 7, separated into right jog and no jog (midline positioned hearts) scored live between 28-30 hpf. Siblings are uninjected controls from same cross as uninjected mutants. N=4, Mean ± SEM.

Table S1. Supplemental information relating *VANGL2* VUS in Tables 1 and 2

Family	Main clinical phenotype	Protein change	Allele count	ClinVar ID	rs ID
1	Holoprosencephaly	S84F	-	nd	rs1571244916
2	Control	R105C	10 / 251194	nd	rs746031069
3	Myelomenigocele	R135W	22 / 247284	nd	rs757382979
4	Diastematomyelia	R177H	-	nd	rs1314974864
5	Control	V178I	18 / 281514	nd	rs146421127
6	Tethered cord				
7	Myelocystocele	L242V	164 / 271770	nd	rs147526057
8	Myelomenigocele				
9	Lipoma of filum terminus	T247M	8 / 270604	nd	rs757874205
10	Fibrolipoma of filum terminus	R270H	3 / 282754	nd	rs573558988
11	Anencephaly, spina bifida	R353C	2 / 250846	9052	rs267607167
12	Anencephaly	F437S	-	nd	rs267607168
13	Caudal agenesis	R482H	31 / 282830	nd	rs149448007
14	New disease	R135W	22 / 247284	nd	rs757382979
15	Heterotaxy	R169H	-	nd	rs1187014622
16	Hearing loss	E465A	66 / 282818	nd	rs143298058

<u>Table S2. Statistical tests relating to Figure 4K.</u>

AP length compared to uninjected *vangl2*^{m209}

Brown-Forsythe and Welch ANOVA with multiple comparisons

**Variant**	**Embryos**	**p value vs. uninjected mutant**	**p value vs. mutant + WT *VANGL2***	**Category**
L242V	40	**** (<0.0001)	ns (0.6357)	Rescue
R135W	40	**** (<0.0001)	ns (<0.9999)	Rescue
R105C	39	**** (<0.0001)	ns (<0.9999)	Rescue
S84F	39	**** (<0.0001)	ns (0.4267)	Rescue
R270H	39	**** (<0.0001)	ns (0.1146)	Rescue
R169H	40	**** (<0.0001)	* (0.0315)	Partial rescue
T247M	40	*** (0.0004)	* (0.0129)	Partial rescue
R353C	36	* (0.0464)	**** (<0.0001)	Partial rescue
V178I	40	ns (0.1839)	**** (<0.0001)	No rescue
R177H	40	ns (0.2299)	**** (<0.0001)	No rescue
R482H	40	** (0.0060)	**** (<0.0001)	Exacerbates
F437S	38	**** (<0.0001)	**** (<0.0001)	Exacerbates
E465A	39	**** (<0.0001)	**** (<0.0001)	Exacerbates

Table S3. Statistical tests relating to Figure 5D.

FBM migration in uninjected controls and WT *VANGL2* rescue

Fisher's exact tests

**Group**	**No migration**	**Migration**	**Embryos**	**p value vs. sibling**	**p value. vs mutant**
Uninjected sibling	0	40	40	-	-
Uninjected *vangl2*^{m209}	40	0	40	**** (<0.0001)	-
vangl2^{m209} + WT	27	13	40	**** (<0.0001)	**** (<0.0001)

Table S4. Statistical tests relating to Figure 5E.

FBM migration compared to uninjected *vangl2*^{m209}

Fisher's exact tests

**Variant**	**Embryos**	**No migration**	**Migration**	**p value vs. uninjected mutant**	**p value vs. mutant + WT *VANGL2***	**Category**
R353C	40	28	12	*** (0.0002)	ns (>0.9999)	Rescue
R270H	40	30	10	** (0.0010)	ns (0.6219)	Rescue
T247M	40	33	7	* (0.0117)	ns (0.1961)	Rescue
R482H	40	37	3	ns (0.2405)	* (0.0103)	No rescue
E465A	40	37	3	ns (0.2405)	* (0.0103)	No rescue
R135W	40	36	4	ns (0.1156)	* (0.0269)	No rescue
S84F	40	36	4	ns (0.1156)	* (0.0269)	No rescue
F437S	40	37	3	ns (0.2405)	* (0.0103)	No rescue
R177H	40	37	3	ns (0.2405)	* (0.0103)	No rescue
R105C	40	36	4	ns (0.1156)	* (0.0269)	No rescue
R169H	40	37	3	ns (0.2405)	* (0.0103)	No rescue
V178I	40	36	4	ns (0.1156)	* (0.0269)	No rescue
L242V	40	38	2	ns (0.4937)	** (0.0031)	No rescue

Table S5. Statistical tests relating to Figure 6C.

Abnormal *spaw* compared to uninjected *vangl2*^{m209}

One-way ANOVA with multiple comparisons

**Variant**	**Embryos (across N=4)**	**p value vs. uninjected mutant**	**p value vs. mutant + WT *VANGL2***	**Category**
T247M	20, 27, 22, 18	ns (0.9992)	*** (0.0002)	No rescue
R135W	21, 22, 22, 32	ns (>0.9999)	**** (<0.0001)	No rescue
R353C	20, 20, 20, 22	ns (>0.9999)	**** (<0.0001)	No rescue
R482H	22, 20, 22, 24	ns (>0.9999)	**** (<0.0001)	No rescue
V178I	27, 25, 22, 23	ns (>0.9999)	**** (<0.0001)	No rescue
R270H	22, 21, 21, 24	ns (>0.9999)	**** (<0.0001)	No rescue
R105C	23, 22, 21, 24	ns (>0.9999)	**** (<0.0001)	No rescue
R169H	19, 25, 21, 24	ns (>0.9999)	**** (<0.0001)	No rescue
L242V	23, 23, 24, 22	ns (0.9943)	**** (<0.0001)	No rescue
E465A	20, 43, 28, 33	ns (0.8051)	**** (<0.0001)	No rescue
R177H	21, 23, 21, 26	ns (0.2904)	**** (<0.0001)	No rescue
F437S	22, 21, 29, 30	* (0.0459)	**** (<0.0001)	Exacerbates
S84F	21, 21, 25, 23	**** (<0.0001)	**** (<0.0001)	Exacerbates

<u>Table S6. Statistical tests relating to Figure 7C.</u>

Abnormal jogging compared to uninjected *vangl2*^{m209}

One-way ANOVA with multiple comparisons

**Variant**	**Embryos (across N=4)**	**p value vs. uninjected mutant**	**p value vs. mutant + WT *VANGL2***	**Category**
R353C	25, 21, 29, 24	* (0.0370)	ns (0.4981)	Rescue
T247M	28, 29, 36, 31	ns (0.0562)	ns (0.3815)	Partial rescue
S84F	25, 26, 25, 26	ns (0.3481)	* (0.0321)	No rescue
V178I	28, 24, 25, 37	ns (>0.9999)	*** (0.0006)	No rescue
R169H	23, 24, 22, 23	ns (>0.9999)	**** (<0.0001)	No rescue
R105C	27, 28, 35, 33	ns (>0.9999)	**** (<0.0001)	No rescue
L242V	26, 23, 22, 32	ns (>0.9999)	**** (<0.0001)	No rescue
R135W	28, 42, 34, 31	ns (>0.9999)	**** (<0.0001)	No rescue
R270H	25, 26, 25, 26	ns (>0.9999)	**** (<0.0001)	No rescue
F437S	25, 32, 26, 26	ns (>0.9999)	**** (<0.0001)	No rescue
R177H	25, 28, 27, 27	ns (>0.9999)	**** (<0.0001)	No rescue
R482H	28, 31, 24, 26	ns (>0.9999)	**** (<0.0001)	No rescue
E465A	28, 26, 26, 25	ns (0.9995)	**** (<0.0001)	No rescue

Table S7. Statistical tests relating to Figure 7E.

Cardia bifida compared to uninjected *vangl2^{m209}*

One-way ANOVA with multiple comparisons

**Variant**	**Embryos (across N=4)**	**p value vs. uninjected mutant**	**p value vs. mutant + WT *VANGL2***	**Category**
T247M	See table S5	ns (>0.9999)	ns (0.3355)	Partial rescue
R353C		ns (>0.9999)	ns (0.0789)	Partial rescue
R270H		ns (0.9990)	** (0.0079)	No rescue
E465A		ns (0.9787)	** (0.0031)	No rescue
F437S		ns (0.9657)	** (0.0025)	No rescue
R169H		ns (0.7080)	*** (0.0005)	No rescue
R177H		ns (0.6355)	*** (0.0004)	No rescue
L242V		ns (0.3452)	*** (0.0001)	No rescue
R105C		ns (0.2553)	**** (<0.0001)	No rescue
R482H		ns (0.2140)	**** (<0.0001)	No rescue
V178I		ns (0.1110)	**** (<0.0001)	No rescue
S84F		ns (0.0642)	**** (<0.0001)	No rescue
R135W		**** (<0.0001)	**** (<0.0001)	Exacerbates

Table S8. Primers for site mutagenesis of the WT *VANGL2* plasmid

**Variant**	**Forward**	**Reverse**
S84F	GAGGTCATCATGGAAGATGCTGTGCTCTGAGGT	ACCTCAGAGCACAGCATCTTCCATGATGACCTC
R105C	CACACCCAGGTGACAGGAGCAGTCCAGAG	CTCTGGACTGCTCCTGTCACCTGGGTGTG
R135W	CCAGCTCCTCCCACCACAGCAGTGG	CCACTGCTTGGTGGGAGGAGCTGG
R169H	CAGCTGGGCTCTGTTCTTCCACCGGCCCAAGGCCTCGCTGC	GCAGCGAGGCCTTGGGCCGGTGGAAGAACAGAGCCCAGCTG
R177H	CAGCACAAAGACGTGGGGCAGCGAGGC	GCCTCGCTGCCCCACGTCTTTGTGCTG
V178I	CGCAGCACAAAGATGCGGGGCAGCGAG	CTCGCTGCCCCGCATCTTTGTGCTGCG
L242V	ACTGAGGCTGGACCTGGCGCAGCTC	GAGCTGCGCCAGGTCCAGCCTCAGT
T247M	CACGACCTTGAGCATGAACTGAGGCTGGAGC	GCTCCAGCCTCAGTTCATGCTCAAGTCGTG
R270H	CCACACTGCCACGTGCTGGATGCTGAG	CTCAGCATCCAGCACGTGGCAGTGTGG
R353C	GCCCTCCTCTTGCACACCCTTCGCTCA	TGAGCGAAGGGTGTGCAAGAGGAGGGC
F437S	AGTATCGCTCCAAGGAGGCCTTGGGCGTC	GACGCCCAAGGCCTCCTTGGAGGCGATACT

E465A	GGTCACCGGCTCCGCGCTCACC AATGT	ACATTGGTGAGCGCGGAGCCGGT GACC
R482H	CAGGCTGAAGTCCTGGTGTTTT AAGAGGAAAACG	CGTTTTCCTCTTAAAACACCAGG ACTTCAGCCTG
